# Psychosocial support for Arabic-speaking refugees residing in Switzerland (Sui app): A mixed-methods randomised controlled trial

**DOI:** 10.1016/j.jmh.2025.100379

**Published:** 2025-11-27

**Authors:** Rilana T Stoeckli, Viktoria Zoellner, Farhad Haji, Monia Aebersold, Sebastian Burchert, Jessica Wabiszczewicz, Christine Knaevelsrud, Eva Heim, Thomas Berger

**Affiliations:** a Department of Clinical Psychology and Psychotherapy, Institute of Psychology, University of Bern, Fabrikstrasse 8 3014 Bern, Switzerland; b Swiss Red Cross, Bern, Switzerland; c Department of Clinical Psychological Intervention, Freie Universität Berlin, Habelschwerdter Allee 45 14195 Berlin, Germany; d Institute of Psychology, University of Lausanne Geopolis, Quartier Mouline 1015 Lausanne, Switzerland

**Keywords:** Digital MHPSS, Quality of life, Cultural adaptation, Refugees, Arabic, Psychosocial

## Abstract

**Introduction:**

Refugees’ quality of life (QOL) is significantly impacted by the challenging post-migration context. Digital mental health and psychosocial support (MHPSS) services offer scalable, culturally adaptable solutions. This study evaluates the Sui app, co-developed with Arabic-speaking refugees in Switzerland to address barriers to mental healthcare and promote psychosocial well-being.

**Methods:**

A mixed-methods randomised controlled trial assessed the app’s impact on QOL and mental health outcomes among Arabic-speaking refugees. Participants were randomised (2:2:1) into app-only (Sui, *n* = 68), app with peer support (Sui+, *n* = 68), or waitlist (WL, *n* = 34). The primary outcome was QOL (WHOQOL-BREF), secondary outcomes included mental health indicators (e.g., depressive, anxiety, post-traumatic stress and somatic symptoms). Quantitative data were collected at baseline, post (8 weeks), and follow-up (16 weeks). Semi-structured interviews explored user experiences. Ethical approval was obtained, and all participants gave informed consent.

**Results:**

Intention-to-treat analyses (*N* = 170) using linear mixed-effects models revealed no significant group-by-time interaction effects, indicating that changes over time did not differ significantly between the three groups for QOL (e.g., psychological domain: *F*(2134.67) = 1.85, *p* = .16) and for any of the secondary outcomes at post-assessment. Small but significant effects emerged at follow-up between active groups. Median app usage for was 13.1 min, with 95 participants successfully logging in. Qualitative findings identified barriers such as unclear onboarding, technical issues, and limited applicability. Nonetheless, participants expressed general satisfaction with the app’s content and peer support.

**Conclusions:**

The Sui app was well-accepted and its context-sensitive design offers promise for digital MHPSS services for refugees. However, lack of significant outcome improvements and limited engagement underscore the need for technical refinements, stronger onboarding, and more visible peer support.

## Highlights

• Evaluation of the Sui SRK app, co-developed with Arabic-speaking refugees in Switzerland.
• The Sui SRK app presents a novel digital approach to psychosocial support.
• No significant improvements in quality of life or mental health outcomes.
• Positive qualitative feedback emphasises the app’s relevance and concept.
• Future research should expand app’s reach to other refugee populations.

## 1 Introduction

The impact of displacement due to war or persecution on vulnerability to mental illness is well documented in the literature (Blackmore et al., 2020; Patanè et al., 2022; Turrini et al., 2019). Adverse events before, during and after migration contribute to an increased incidence of psychopathological symptoms (Abubakar et al., 2018). In addition, post-migration stressors, often referred to as “Post-Migration Living Difficulties” (PMLD), are highly prevalent among refugees. In Switzerland, these most commonly include difficulties with employment, government regulations, housing, education, finances and language (Drescher et al., 2021; Kiselev et al., 2020). PMLDs can also include worries about family back home, isolation or concerns about not having access to the health system (Aragona et al., 2012). PMLDs have a negative impact on mental health, either directly (Gleeson et al., 2020; Hynie, 2018; Jannesari et al., 2020) or by disrupting interdependent psychosocial pillars necessary for recovery and resilience.

These pillars are outlined in the Adaptation and Development after Persecution and Trauma (ADAPT) model by Silove et al. (Silove, 2013) and defined as safety/security, bonds/networks, justice, roles/identity, and existential meaning. They have been described to provide a holistic view of what is needed to comprehensively support conflict-affected populations. Quality of life (QOL) as another holistic concept links past experiences, ongoing psychosocial challenges and health. The World Health Organization (WHO) defines QOL using a multidimensional approach, categorizing it into physical, psychological, social, and environmental domains (Skevington et al., 2004; WHOQOL-Group 1998). The concept includes mental health but also takes the individual’s current living context into account.

QOL has consistently been reported to be lower among refugees in low-, middle- and high-income countries compared to the general population (Al Masri et al., 2021; Gagliardi et al., 2021; Leiler et al., 2019; Sengoelge et al., 2022). Social factors have been shown to be strongly associated with health-related QOL (Correa-Velez et al., 2020; Haj-Younes et al., 2020; Schick et al., 2016; van der Boor et al., 2020). For instance, unemployment and the presence of very few family members predicted poorer QOL in a study with Iraqi refugees in Jordan (Al-Smadi et al., 2017). Psychopathology, such as anxiety, has also been reported to predict lower QOL (Al-Smadi et al., 2017) or to be associated with lower QOL (Buchcik et al., 2023; Leiler et al., 2019). Finally, stressors specific to the post-migration context further mediate the association between potentially traumatic events and QOL (Dangmann et al., 2021).

Given the significant vulnerability to mental health issues and associated socio-structural challenges commonly observed in post-migration contexts, it is essential to broaden the reach of mental healthcare services to a wider population of people in need (Koesters et al., 2018; Turrini et al., 2019). Mental Health and Psychosocial Support (MHPSS) reflects a comprehensive approach, addressing both mental health disorders – such as trauma-related conditions – and psychosocial challenges linked to cultural and contextual factors (Inter-Agency Standing Committee Task Force on Mental Health 2007; Weine et al., 2002). Low-threshold psychosocial support, a key component of MHPSS, is designed to be accessible and inclusive, targeting a diverse population with both basic and focused interventions. As outlined by the International Federation of the Red Cross (IFRC) Reference Centre for Psychosocial Support (2020), such services aim to promote positive mental health, enhance psychosocial wellbeing, and provide prevention activities. Regarding traditional mental healthcare, a study with Syrian refugees in Switzerland identified several structural and sociocultural access barriers, underscoring the limitations of specialised treatments in meeting the needs of diverse populations (Kiselev, 2020).

Digital interventions, with their potential for adaptability, scalability, effectiveness, and accessibility, represent an innovative approach to supporting refugees in need (Ashfaq et al., 2020). Studies indicate that app-supported smartphone interventions are generally effective in improving QOL (Linardon et al., 2019). Promising results were also reported in improving QOL of refugees through digital methods (Nascimento et al., 2023). Furthermore, the widespread use of digital devices within refugee communities indicates that technology-based interventions are viable and practical (Alencar et al., 2019).

A common challenge with digital interventions is low adherence, with many users disengaging before completing the program (Andersson and Berger, 2021). Research shows that interventions with human support achieve higher adherence rates (Musiat et al., 2022) and better outcomes (Koelen et al., 2022). Providing human support to refugees presents unique challenges, as facilitators need to speak the refugees' native languages and understand their life situation. A potential solution to this barrier is task-shifting, for example through peer support, where trained non-specialists with similar backgrounds or experiences provide additional support to complement the intervention. This approach has been successfully tested with refugees and asylum seekers in face-to-face interventions (Turrini et al., 2019) and digital mental health interventions (El-Haj-Mohamad et al., 2023), although adherence remained a challenge.

Given the low QOL and significant mental healthcare barriers encountered by numerous refugees, this study introduces the "Sui app" (Selfhelp, Support, Information) as a digital MHPSS service designed to provide accessible, culturally sensitive psychosocial support. The objective of this study is to evaluate the app's impact on QOL and mental health outcomes among Arabic-speaking refugees in Switzerland, both with and without additional peer support. Furthermore, the study assesses the app’s acceptability and explores modifications for the use in a future version. To our knowledge, no digital intervention or service has been evaluated that combines specific, country-relevant information with low-threshold psychological support. Such an approach represents not only a crucial advancement in providing accessible, culture-sensitive services tailored to the living context of refugees but also contributes to the limited body of evidence on psychosocial support.

## 2 Materials and methods

### 2.1 Study design

This mixed-methods study was a parallel three-arm randomised controlled trial (RCT). Participants were randomly assigned to one of three groups: (1) Sui: app access only, (2) Sui+: app access with weekly peer support, and (3) WL: waitlist control group with delayed app access. The primary outcome was assessed at 8 weeks (post-treatment) and participants were followed up to 16 weeks. Participants were invited to join an optional qualitative telephone interview to reflect on their experience with the app.

This study was preregistered on clinicaltrials.gov (NCT05651737) and conducted in accordance with the Declaration of Helsinki. It was approved by the Cantonal Ethics Committee of Bern (CEC; ID: 2022–00,607). Participants paper signed an informed consent before entering the study.

### 2.2 Study groups: Sui, Sui+, and waitlist

**Fig. 1:**
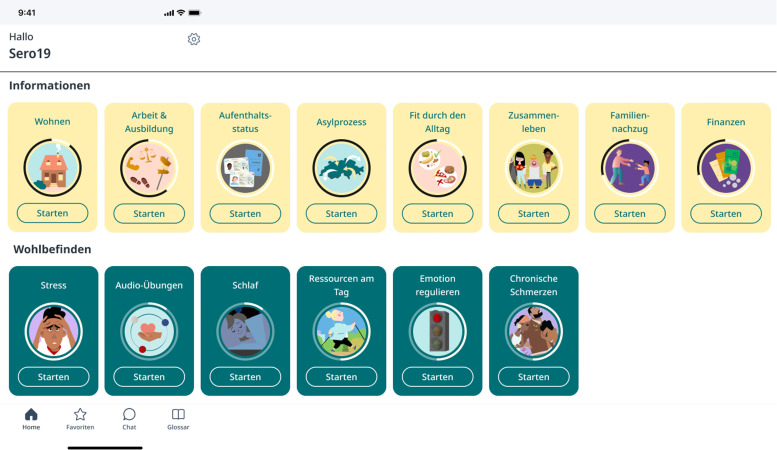
Sui SRK app’s two main chapters: Information: Housing, Work & Education, Residence status, Asylum process, Health promotion, Social living, Family reunification, Finances; Well-being: Stress, Audio exercises, Sleep, Resources, Emotion regulation, Chronic pain.

The Sui app was co-developed bottom-up in a participatory process with experts and representatives of the target group, as described in detail by Stoeckli et al. (Stoeckli et al., 2025). The documentation follows the cultural adaptation framework of Heim et al. (Heim et al., 2021). The app contains four introductory general chapters, nine chapters with practical information about life in Switzerland and five chapters on psychological well-being, featuring psychoeducation, coping skills, and exercises (see Fig. 1). It offers content in various formats (text, videos, audio, illustrations) and is designed for non-linear access, as needed.

The Sui+ group received weekly personalised support messages in Arabic from a peer via in-app chat. They were written based on templates and focused on promoting well-being. Peers guided their assigned users through the app, suggested relevant exercises, and referred to chapters in the app for specific concerns, and answered questions within 48 h. The Sui group used the app without support. Participants in both active groups received automatic push notification reminders after 5 and 10 days of inactivity. The WL received delayed access to the Sui app without support 8 weeks after randomisation.

### 2.3 Sample size and power

The sample size was determined a priori using G*Power 3 (Faul et al., 2007) to detect a small-to-medium effect (*f* = 0.15) between the active groups and a medium effect (*f* = 0.25) between the active groups and the WL on the primary outcome (QOL) at the post-assessment. We assumed correlations of *r* = 0.5 within participants between pre- and post-intervention measurements and a power (1 – β) of 0.80. This required a minimum of 60 participants per active group and 30 participants for the WL, totalling 150 participants. For qualitative data collection, it was decided to interview everyone willing to participate.

### 2.4 Recruitment, selection of participants and procedure

Recruitment occurred between October 2022 and November 2023, using flyers and posters, snowball sampling, social media, personal networks, and visits to cantonal asylum centres with the help of an Arabic-speaking translator.

After registering online and providing signed consent, participants completed a baseline self-assessment via an emailed link. Inclusion criteria: Participants had to be 18 years or older, speak Arabic as a native or secondary language, have sufficient Arabic literacy, reside in Switzerland for 5 years or less, and have access to a smartphone and the internet. Participants were excluded if they indicated acute suicide risk (PHQ-9 suicide item) or reported a diagnosis of psychosis or bipolar disorder.

If eligible, participants were block-randomised into one of three groups (1) Sui, (2) Sui+, or (3) WL, using a 2:2:1 concealed allocation ratio. Randomisation was implemented via the data collection and management capture tool REDcap (Harris et al., 2019). Immediate app access information was provided to participants in the active groups, with the Sui+ group receiving their first peer support message within two days. Each participant received two subsequent links to self-assessments: one after 8 weeks (post-assessment) and another after 16 weeks (follow-up). They were remunerated with a 20-franc supermarket voucher for each completed assessment up to a maximum of 60 Swiss francs.

In the post assessment (active groups), and in the follow-up assessment (WL), participants were asked if they wished to participate in the qualitative interview. Interview appointments were scheduled 2–9 weeks after participants expressed their interest. No additional incentives were offered for interview participation.

### 2.5 Quantitative measures

Sociodemographic variables (e.g., gender, age, nationality, and education level), along with religious affiliation and activity, chronic illness, lifetime diagnosis of bipolar affective or psychotic disorder, and recruitment source, were self-assessed in the questionnaire at baseline. Further socio-demographic variables (e.g. employment status, living situation, marital status) were self-reported at all three timepoints.

#### 2.5.1 Primary outcomes

The primary outcome was QOL, measured using the 24-item WHOQOL-BREF questionnaire (Skevington et al., 2004; WHOQOL-Group 1998) in its validated Arabic version with Cronbach’s α values ≥0.7 for all domains except for the social relations domain (α = 0.69) (Ohaeri and Awadalla, 2009). Items are categorised into four domains: physical health (7 items), psychological health (6 items), social relations (3 items), and environment (8 items). Items for physical health contain questions about pain, energy, sleep, mobility, activities, medication, and work. Psychological health items cover positive feelings, thinking, self-esteem, body image, negative feelings, and spirituality. Social items query satisfaction with current relationships, perception of support, and sex life, with response to the last item being optional. Environment items cover questions about safety, housing, finances, services, information accessibility, natural environment, and transportation possibilities. Items are rated on a five-point Likert scale from 1–5. Scores are transformed to a 4–20 scale for each domain, with higher scores reflecting a better QOL. An overall score is not recommended by the WHO (WHOQOL-Group 1998). In the current sample, Cronbach's α was satisfactory with 0.76 for the physical, 0.74 for the psychological and 0.83 for the environment domain at pre-treatment, as well as a questionable internal consistency of α = 0.61 for the social relations domain.

#### 2.5.2 Secondary outcomes

Secondary outcomes included the following: Depressive symptoms were measured with the 9-item Patient Health Questionnaire-9 (PHQ-9, (Kroenke et al., 2001), validated Arabic version with Cronbach’s α = 0.88 (Sawaya et al., 2016); and good internal consistency in the current sample, α was 0.86). Anxiety symptoms were assessed with the 7-item Generalised Anxiety Disorder-7 (GAD-7, (Löwe et al., 2008), validated Arabic version with Cronbach’s α = 0.95 (Sawaya et al., 2016) and good internal consistency, α = 0.89, in the current sample). Posttraumatic symptoms were assessed with the Posttraumatic Stress Disorder Checklist (PCL-5, validated Arabic version with Cronbach’s α = 0.85 (Ibrahim et al., 2018), shortened validated 8-item version (Price et al., 2016)). Internal consistency in the current sample was good, with α = 0.92. Somatic symptoms were measured with the PHQ-15 (Kroenke et al., 2002, validated Arabic version with Cronbach’s α = 0.83 (AlHadi et al., 2017), and in the current sample good internal consistency with α = 0.85)). PMLDs were evaluated using the PMLD-Checklist (PMLD-CL, Silove et al., 1997)), adapted for Switzerland and translated to Arabic by Schick et al. (2016). In the current sample, internal consistency was good with α = 0.84. Self-stigma was measured with two sections of the Self-Stigma of Mental Illness Scale - Shortform (SSMIS-SF), assessing stereotype awareness and stereotype agreement. Each subscale consisted of five items (Cronbach’s α ≥ 0.72, (Corrigan et al., 2012)). The Arabic translation was adopted from another self-help app study (Röhr et al., 2021), and Cronbach’s α in the current sample were poor with 0.24 (awareness) and 0.37 (agreement). See Appendix B.2 for an overview of internal consistency values for all measures in the current sample.

#### 2.5.3 Further quantitative measures

The client satisfaction questionnaire (CSQ-8, (Attkisson and Zwick, 1982)), which was adapted to the Sui app and translated to Arabic by the study team’s translator (FH), was assessed 8 weeks after app access. App usage was measured by timestamps from each app use, providing information about the intensity of app usage during the 8-week active study phase.

### 2.6 Qualitative measures

For qualitative data, semi-structured telephone interviews were conducted in English, in German, or with simultaneous Arabic-German translation. Aim of the interviews was to gather feedback on user experience, suggestions for improvement, and current needs. After 12 interviews, the first guide was adapted to a shorter second version. The second version took into account the participant’s app usage time and their answers to the satisfaction questionnaire (see Appendix A.1 and A.2 for interview guides). All interviews were conducted by psychology master’s students.

### 2.7 Analyses

#### 2.7.1 Quantitative analyses

As per the intention-to-treat (ITT) principle, we included all randomised participants in the analyses. For the primary analysis, we utilised the lmer function from the lme4 package (Bates et al., 2015) in R (version 4.4.1) to conduct linear mixed models with restricted maximum likelihood estimation, evaluating changes in the primary and secondary outcome variables between the baseline and post-assessment. The fixed effects included timepoint (baseline, post), study group (Sui, Sui+, waitlist) and their interaction. The same model was used to assess the effects on the secondary outcome measures. The follow-up analyses included the third timepoint (follow-up), excluding the WL group data, as they gained access to the app after the post-assessment.

To evaluate group differences at baseline, we used ANOVAs for continuous data and Chi-squared tests for nominal data. At post-assessment we examined differences between the active groups for satisfaction and app usage time. Where relevant assumptions for the respective tests were violated (e.g., normality), we conducted non-parametric tests such as Fisher’s Exact Test, Kruskal-Wallis Test or Wilcoxon Rank-Sum Test.

#### 2.7.2 Qualitative analyses

Qualitative analyses followed Mayring's (2015) summarising content analysis for its rules-based approach and allowing for the integration of quantitative elements. Interviews were inductively coded from simplified transcripts to reduce texts with equal meaning into categories. After coding four interviews, the coding team refined the categories and then continuously updated them as new codes emerged. Subsequently, all categories were clustered into main categories. All interviews were revised in the end using the final category system to check compliance. The coding team included three master’s students in psychology (who also conducted the interviews) and this article’s first author RS, a PhD student in clinical psychology.

## 3 Results

### 3.1 Sample

**Fig. 2:**
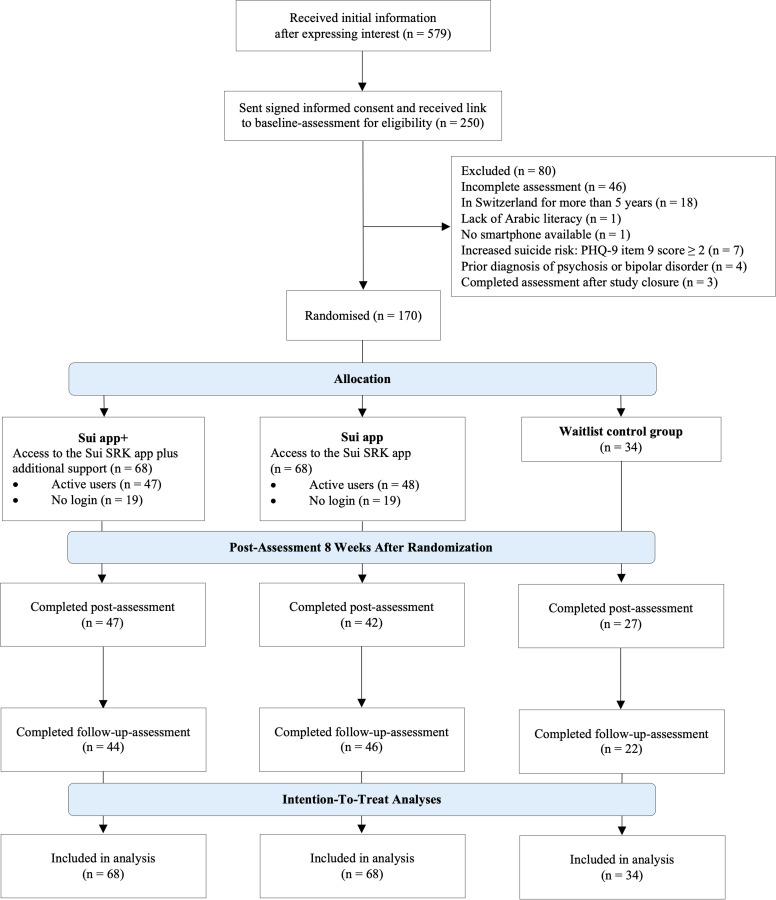
Participants’ study flow.

Participant flow of the study is displayed in Fig. 2. The final sample size was 170, 68 per active group, 34 in the WL. The sample consisted of 95 males (55.9 %), 74 females (43.5 %), and one non-binary individual (0.6 %). The age range was between 18 and 63 years, with a mean age of *M* = 32.88 (*SD* = 9.52). The most effective recruitment strategy was visits to asylum centres, accounting for 58 participants (34.1 %), followed by 48 participants (28.2 %) who heard about the study through friends, 35 (20.6 %) through social media, 14 (8.2 %) via flyers or posters, 10 (5.9 %) through professional referrals, and 5 (2.9 %) by other means. Participants primarily came from German-speaking (78.2 %), followed by French-speaking (21.2 %), and Italian-speaking (0.6 %) Switzerland.

A total of 58 participants volunteered for the interviews. Of these, 11 could not be reached and 6 were solely contacted for login-related questions because they had not logged in. The final sample included 41 participants (23 males, 18 females) from ten different countries with a majority from Syria (*n* = 27). The average age across participants was *M* = 36.22, *SD* = 9.58. Most of the interviews were conducted in Arabic with German translation, two were held in German and one in English. Duration was 50–75 min for the first 12 and 15–36 min for the remaining 29 interviews. Group distribution included 19 participants from Sui+ (7 of whom were non-responsive to support messages), 17 from Sui, and 5 from the WL. Information on individual age, app usage, gender, and study group are displayed in Appendix D.2.

### 3.2 Baseline comparisons

Baseline sociodemographic characteristics of the three study groups are presented in Table 1. There were no baseline between-group differences on the primary or secondary outcomes, except for the psychological domain of QOL (*χ^2^* (2) = 8.76, *p* = .01) (for details see Appendix B.1).

**Table 1:** Participant sample at baseline and comparisons between study groups.

Characteristics	Sui+(n = 68)	Sui (n = 68)	Waitlist (n = 34)
Mean age, years M (SD)	33.0 (9.40)	33.4 (10.13)	31.6 (8.63)
Age range	18–53	18–63	19–60
Gender, n ( %)			
Male	36 (53.0)	44 (64.7)	15 (44.1)
Female	32 (47.1)	24 (35.3)	18 (53.0)
Non-binary	0 (0.0)	0 (0.0)	1 (2.9)
Residence status, n ( %)			
B Refugee	23 (33.82)	30 (44.12)	15 (44.11)
B / F Foreigner	9 (13.24)	5 (7.35)	7 (20.588)
F Refugee	8 (11.76)	6 (8.82)	4 (11.76)
N Asylum seeker	25 (36.76)	20 (29.41)	5 (14.71)
Other	3 (4.11)	6 (8.82)	3 (8.82)
Nationality, n ( %)			
Syria	51 (75.00)	38 (55.88)	21 (61.76)
Iraq	4 (5.88)	4 (5.88)	3 (8.82)
Egypt	0 (0.00)	2 (2.94)	4 (11.76)
Palestine	3 (4.41)	5 (7.35)	1 (2.94)
Other	10 (14.71)	19 (27.94)	5 (14.71)
Religion, n ( %)			
Sunni	53 (77.94)	56 (82.35)	26 (76.47)
Other	15 (22.06)	12 (17.65)	8 (23.53)
Religiousness, n ( %)			
Low	29 (42.65)	21 (30.88)	11 (32.35)
Medium	30 (44.12)	31 (45.59)	19 (55.88)
High	9 (13.24)	16 (23.53)	4 (11.76)
Currently in psychotherapy, n ( %)			
Yes	13 (19.12)	5 (7.35)	4 (11.76)
No	55 (80.88)	63 (92.65)	30 (88.24)
Time in Switzerland, years M (SD)	1.62 (1.36)	1.95 (1.49)	1.66 (1.9)
Living situation, n ( %)			
Federal asylum centre	11 (16.18)	6 (8.82)	4 (11.76)
Cantonal asylum centre	23 (33.82)	23 (33.82)	11 (32.35)
Apartment	29 (42.65)	34 (50.0)	18 (52.94)
Shared apartment	5 (7.35)	5 (7.35)	1 (2.94)
Highest educational level, n ( %)			
No school	9 (13.24)	7 (10.30)	4 (11.76)
1–12 years of school	22 (32.35)	14 (20.59)	7 (20.59)
Maturity / Apprenticeship	11 (16.18)	16 (23.53)	10 (29.41)
University / College	26 (38.24)	31 (45.59)	13 (38.24)
Marital status, n ( %)			
Single	20 (29.41)	26 (38.24)	11 (32.35)
In a partnership	0 (0.00)	2 (2.94)	1 (2.94)
Married	42 (61.76)	36 (52.94)	19 (55.88)
Divorced	5 (7.35)	1 (1.47)	1 (2.94)
Widowed	1 (1.47)	3 (4.41)	1 (2.94)
Employment, n ( %)			
Full or part-time job	6 (8.82)	5 (7.35)	5 (14.71)
Unemployed	33 (48.53)	27 (39.71)	6 (17.65)
Househusband / Housewife	7 (10.29)	7 (10.29)	8 (23.53)
In training / Other	22 (32.35)	29 (42.65)	13 (38.24)

Note. Descriptive statistics are reported as means (SD) or counts ( %). No statistical comparisons

were conducted due to small cell sizes.

### 3.3 Study dropout analysis

Data for the primary outcome were missing for 54 participants at post-assessment (31.8 %, Sui+, *n* = 47; Sui, *n* = 42; WL, *n* = 27) and for 58 participants at follow-up (34.1 %, Sui+, *n* = 44; Sui, *n* = 46; WL, *n* = 22), classifying them as “non-completers” regarding study completion. Completers were older (*M* = 33.9 *SD* = 9.67) than non-completers (*M* = 30.6 *SD* = 8.85; *W* = 3792, *p* = .027). A higher percentage of males dropped out (*χ^2^* (1) = 4.175, *p* = .04). Furthermore, there was a significant relation between residence status and completion (*χ^2^* (5) = 11.17, *p* 0.048) and living situation and completion (*χ^2^* (3) = 8.85, *p* 0.03), though pairwise comparisons revealed no significant differences (all adjusted *p*-values > 0.05).

No differences were found in psychotherapy attendance, nationality, religion, religiousness, time in Switzerland, education level, marital status, or job situation between completers and non-completers. They did not differ on the primary and most of the secondary outcomes at baseline. A significant difference was found only for the stigma awareness outcome (SSMIS-SF Section 1), with completers scoring higher at baseline than non-completers (*W* = 3729.5, *p* = .03).

### 3.4 Effects on the primary and secondary outcomes

**Fig. 3:**
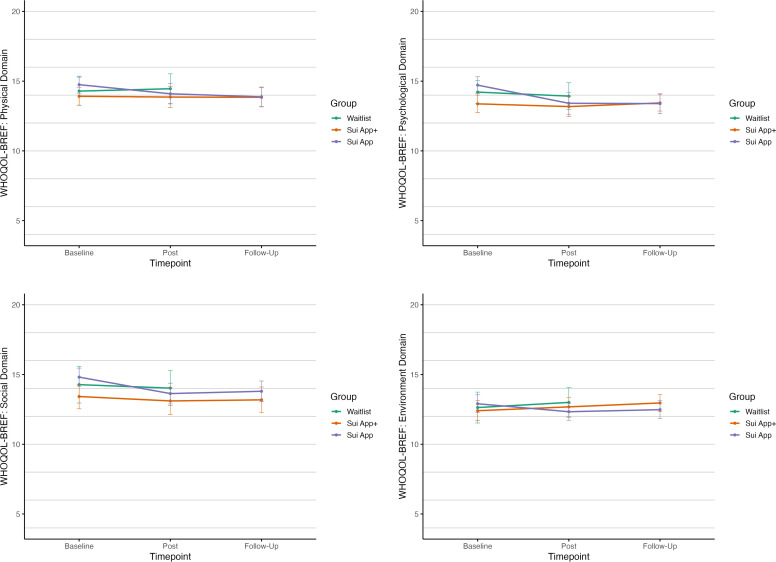
Observed means of the WHOQOL-BREF questionnaire domains at baseline, post and follow-up timepoints, with 95 % confidence intervals. Follow-up mean for the waitlist group is not displayed as it was not controlled.

Baseline and 8-week post-assessment means for all four domains of the WHOQOL-BREF, across the two active groups (Sui+ and Sui) and the WL, are displayed in Fig. 3. Since the WL gained access to the app after post-assessment, the 16-week follow-up means are not controlled and therefore not displayed.

Table 2 presents observed means for baseline, post-assessment (including estimated means), and follow-up. Effect sizes for within- and between-group differences and overall effects of the group-by-time interaction are also displayed in Table 2. No significant group-by-time interaction effects were found for any of the linear mixed models at the 8-week post-assessment for primary or secondary outcomes (p-values > 0.05; see Appendix C for the complete outcome table across all three timepoints). For the two active groups only, the linear mixed model showed a statistically significant group-by-time interaction effect on the psychological domain of QOL, *F*(2193.47) = 5.06, *p* = .01 at 16-week follow-up with an effect size of Cohen’s *d* = 0.02. Similarly, for SSMIS-SF stigma agreement, the model yielded a significant effect at 16-week follow-up, *F*(2, 192.9) = 3.50, *p* = .03 (*Cohen’s d* = 0.12).

**Table 2:** Observed and estimated means at baseline and post-assessment, and within- and between-group effect sizes (ITT sample) at post-assessment.

Measure	Study group	Baseline		Post (observed)		Post (estimated)		Follow-up (observed)		Baseline-post within-group effect sizes (estimated post means)	Group-by-time interaction (baseline, post)	Between-group effect sizes at post (estimated means)
		Mean (SD)	n	Mean (SD)	n	Mean (SE)	n	Mean (SD)	n	Cohen’s d [95 % CI]	F and df	Cohen’s d [95 % CI]
↑ WHOQOL-BREF mean, 4–20 scale range Physical Domain	Sui+	13.92 (2.67)	68	13.86 (3.07)	47	13.79 (0.37)	68	13.84 (2.85)	44	0.01 [−0.33; 0.34]	F _(2, 128.27)_ = 0.26 p = .77	Sui+ vs. WL: −0.17 [−0.58; 0.24]
	Sui	14.75 (2.52)	68	14.10 (3.01)	42	14.41 (0.39)	68	13.88 (2.91)	46	0.02 [−0.31; 0.36]		Sui+ vs. Sui: −0.20 [−0.54; 0.14]
	WL	14.29 (2.88)	34	14.46 (3.07)	27	14.31 (0.51)	34	14.78 (2.93)	22	−0.00 [−0.48; 0.47]		Sui vs. WL: 0.03 [−0.38; 0.44]
↑ WHOQOL-BREF mean, 4–20 scale range Psychological Domain	Sui+	13.37 (2.60)	68	13.18 (2.96)	47	13.17 (0.37)	68	13.44 (2.40)	44	0.01 [−0.32; 0.35]	F _(2, 134.67)_ = 1.85 p = .16	Sui+ vs. WL: −0.29 [−0.70; 0.12]
	Sui	14.72 (2.47)	68	13.41 (3.25)	42	13.66 (0.38)	68	13.39 (2.92)	46	0.07 [−0.26; 0.41]		Sui+ vs. Sui: −0.16 [−0.50; 0.18]
	WL	14.22 (2.36)	34	13.93 (2.74)	27	14.03 (0.50)	34	14.45 (2.54)	22	0.02 [−0.46; 0.49]		Sui vs. WL: −0.12 [−0.53; 0.29]
↑ WHOQOL-BREF mean, 4–20 scale range Social Relations Domain	Sui+	13.42 (3.63)	68	13.11 (4.00)	47	13.24 (0.47)	68	13.18 (3.82)	44	0.01 [−0.33; 0.34]	F _(2, 125.92)_ = 0.86 p = .42	Sui+ vs. WL: −0.18 [−0.59; 0.24]
	Sui	14.81 (2.68)	68	13.63 (3.08)	42	13.86 (0.49)	68	13.80 (3.01)	46	0.06 [−0.28; 0.40]		Sui+ vs. Sui: −0.16 [−0.50; 0.18]
	WL	14.27 (3.78)	34	14.02 (3.61)	27	13.90 (0.63)	34	14.42 (3.51)	22	0.02 [−0.45; 0.50]		Sui vs. WL: −0.01 [−0.42; 0.40]
↑ WHOQOL-BREF mean, 4–20 scale range Environment Domain	Sui+	12.40 (2.95)	68	12.68 (2.77)	47	12.60 (0.37)	68	12.96 (2.52)	44	−0.01 [−0.35; 0.32]	F _(2, 120.67)_ = 0.56 p = .57	Sui+ vs. WL: −0.05 [−0.46; 0.36]
	Sui	12.90 (2.65)	68	12.33 (2.54)	42	12.69 (0.39)	68	12.48 (2.67)	46	0.01 [−0.32; 0.35]		Sui+ vs. Sui: −0.03 [−0.37; 0.31]
	WL	12.63 (3.18)	34	13.00 (3.05)	27	12.75 (0.51)	34	13.27 (2.09)	22	−0.01 [−0.48; 0.47]		Sui vs. WL: −0.02 [−0.43; 0.39]
↓ PHQ-9 sum, 0–27 range Depressive symptoms	Sui+	8.91 (5.53)	68	9.04 (6.82)	46	9.34 (0.77)	68	9.45 (5.63)	42	−0.01 [−0.34; 0.33]	F _(2, 125.79)_ = 0.68 p = .51	Sui+ vs. WL: 0.39 [−0.02; 0.81]
	Sui	7.99 (5.16)	68	9.74 (6.68)	42	9.43 (0.80)	68	7.76 (4.64)	45	−0.03 [−0.37; 0.31]		Sui+ vs. Sui: −0.01 [−0.35; 0.32]
	WL	6.56 (4.53)	34	6.96 (5.68)	26	7.08 (1.05)	34	6.86 (6.16)	22	−0.02 [−0.49; 0.46]		Sui vs. WL: 0.37 [−0.05; 0.78]
↓ PCL-5 sum, 0–32 range 8-item version Posttraumatic stress disorder symptoms	Sui+	12.16 (7.98)	68	11.67 (9.00)	46	12.39 (1.10)	68	11.83 (8.31)	42	−0.00 [−0.34; 0.33]	F _(2, 121.22)_ = 1.39 p = .25	Sui+ vs. WL: 0.50 [0.08; 0.91]
	Sui	10.31 (7.63)	68	11.64 (9.19)	42	11.17 (1.12)	68	10.80 (7.98)	45	−0.02 [−0.36; 0.32]		Sui+ vs. Sui: 0.13 [−0.20; 0.47]
	WL	9.53 (7.99)	34	7.38 (7.34)	26	8.00 (1.50)	34	7.73 (7.67)	22	0.05 [−0.43; 0.52]		Sui vs. WL: 0.35 [−0.06; 0.77]
↓ GAD-7 sum, 0–21 range Anxiety symptoms	Sui+	6.72 (4.80)	68	7.02 (5.89)	46	7.19 (0.68)	68	7.12 (5.56)	42	−0.02 [−0.35; 0.32]	F _(2, 132.18)_ = 2.34 p = .10	Sui+ vs. WL: 0.54 [0.12; 0.96]
	Sui	5.22 (4.39)	68	7.07 (6.04)	42	6.79 (0.70)	68	5.78 (4.94)	45	−0.06 [−0.40; 0.28]		Sui+ vs. Sui: 0.07 [−0.27; 0.41]
	WL	4.88 (4.01)	34	4.08 (4.63)	26	4.24 (0.92)	34	4.32 (4.11)	22	0.04 [−0.37; 0.46]		Sui vs. WL: 0.45 [0.04; 0.87]
↓ PHQ-15 sum, 0–30 range Somatic symptoms	Sui+	8.55 (5.56)	68	9.07 (6.80)	46	9.03 (0.75)	68	10.00 (6.38)	42	−0.01 [−0.35; 0.32]	F _(2, 123.97)_ = 1.74 p = .18	Sui+ vs. WL: 0.41 [−0.00; 0.83]
	Sui	6.90 (5.19)	68	9.01 (6.54)	42	8.46 (0.77)	68	8.78 (6.40)	45	−0.05 [−0.39; 0.28]		Sui+ vs. Sui: 0.09 [−0.24; 0.43]
	WL	6.65 (4.85)	34	5.80 (5.73)	26	6.53 (1.03)	34	5.98 (4.26)	22	0.01 [−0.47; 0.48]		Sui vs. WL: 0.31 [−0.10: 0.72]
↓ SSMIS-SF 1–9 mean range Section 1: Stigma awareness	Sui+	3.94 (1.70)	68	3.60 (2.15)	46	3.52 (0.26)	68	3.63 (1.47)	41	0.04 [−0.29; 0.38]	F _(2, 138.49)_ = 0.63 p = .54	Sui+ vs. WL: 0.03 [−0.38; 0.45]
	Sui	3.54 (1.71)	68	3.67 (1.78)	42	3.53 (0.27)	68	3.70 (1.86)	46	0.00 [−0.33; 0.34]		Sui+ vs. Sui: −0.00 [−0.34; 0.33]
	WL	3.83 (1.57)	33	3.44 (1.99)	27	3.45 (0.34)	34	3.51 (1.95)	22	0.06 [−0.42; 0.53]		Sui vs. WL: 0.04 [−0.37; 0.45]
↓ SSMIS-SF 1–9 mean range Section 2: Stigma agreement	Sui+	3.72 (1.65)	68	3.00 (1.75)	46	3.00 (0.24)	68	3.47 (1.63)	42	0.07 [−0.26; 0.41]	F _(2, 135.13)_ = 2.61 p = .08	Sui+ vs. WL: −0.17 [−0.58; 0.25]
	Sui	3.21 (1.81)	68	3.44 (1.63)	42	3.29 (0.25)	68	3.38 (1.84)	45	−0.01 [−0.34; 0.33]		Sui+ vs. Sui: −0.14 [−0.48; 0.19]
	WL	3.53 (1.64)	34	3.33 (1.87)	27	3.32 (0.32)	34	3.33 (1.93)	22	0.03 [−0.45; 0.51]		Sui vs. WL: −0.02 [−0.43; 0.40]
↓ PMLD-CL 0–68 range Post-migration living difficulties	Sui+	30.09 (12.54)	68	28.46 (13.51)	46	29.08 (1.81)	68	29.23 (13.05)	43	0.01 [−0.32; 0.35]	F _(2, 127.30)_ = 1.02 p = .36	Sui+ vs. WL: 0.46 [0.04; 0.88]
	Sui	28.10 (13.58)	68	28.40 (14.33)	42	27.90 (1.86)	68	26.63 (13.12)	46	0.00 [−0.33; 0.34]		Sui+ vs. Sui: 0.08 [−0.26; 0.42]
	WL	26.21 (11.96)	34	22.52 (15.06)	27	22.41 (2.43)	34	26.68 (16.27)	22	0.08 [−0.40; 0.55]		Sui vs. WL: 0.37 [−0.04; 0.79]

Notes. WHOQOL-BREF = World Health Questionnaire Quality of Life Short Version, PHQ-9 = Depression Module of Patient Health Questionnaire, PCL-5 = Posttraumatic Stress Disorder Checklist for DSM-5, GAD-7 = Generalized Anxiety Disorder Screening, PHQ-15 = Somatic Module of Patient Health Questionnaire, SSMIS-SF = Self-Stigma of Mental Illness Scale Short Form, PMLD-CL = Post-Migration Living Difficulties Checklist. The symbol ↑ indicates that higher values mean better health, symbol ↓ indicates that lower values mean better health.

### 3.5 App usage

**Fig. 4:**
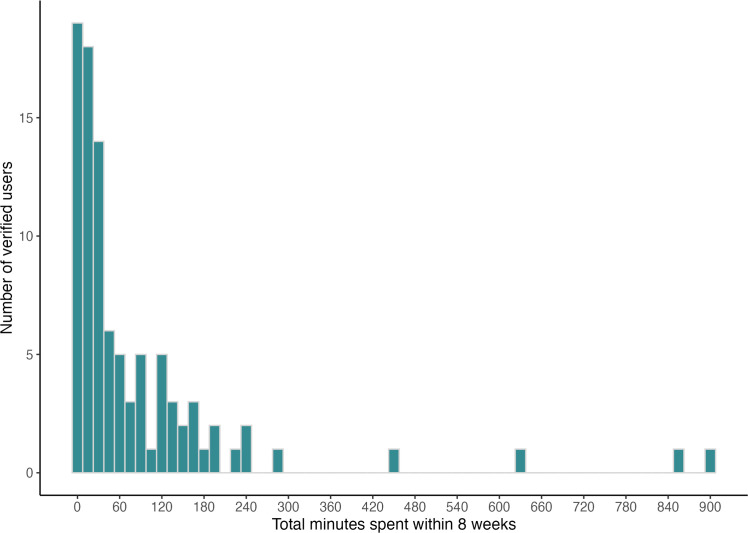
Distribution of app use time of “verified users”, i.e. people who have successfully logged into the Sui app (n = 95).

Data were unavailable for three active groups participants (2.2 %) because they deleted their accounts prior to the study end. For the remaining 133 active participants, the median usage time after 8 weeks was 13.1 min. The mean time spent in the app did not differ between the Sui+ (*M* = 89.95, *SD* = 174.72) and Sui group (*M* = 32.29, *SD* = 56.22) (*W* = 2698.5, *p* = .09). In the Sui+ group, 32 participants (47.1 %) did respond to the peer support. A total of 23 participants (16.9 %) from the active groups never logged in, and 10 participants (7.4 %) had an activated status but no subsequent time stamps. In total, 95 participants (69.9 %) logged in and used the app for at least one minute, indicating a successful login (“verified users”). The median usage time of verified users after 8 weeks was 34.5 min. Fig. 4 shows the distribution of these 95 verified users. Regarding the content, out of all 136 participants from the active study groups, 47 (34.6 %) accessed at least one of the six psychological chapters, 69 (50.7 %) at least one of the nine information chapters, and 51 (37.5 %) at least one of the introductory chapters. A chapter was considered accessed if at least one subchapter was completed. The median usage time of the 41 participants who participated in the interviews was 31.0 min 8 weeks after receiving access to the app.

### 3.6 Participant satisfaction

The average score for satisfaction with the app (CSQ-8) at post-assessment was at *M* = 2.84 (*SD* = 0.55, *n* *=* 46) for the Sui+ group and at *M* = 2.81 (*SD* = 0.57, *n* = 41) for the Sui app group. On a scale of 1–4, these scores indicate overall satisfaction with the app. The two active groups did not differ significantly regarding their satisfaction scores (*W* = 1002, *p* = .62).

### 3.7 Qualitative results

**Fig. 5:**
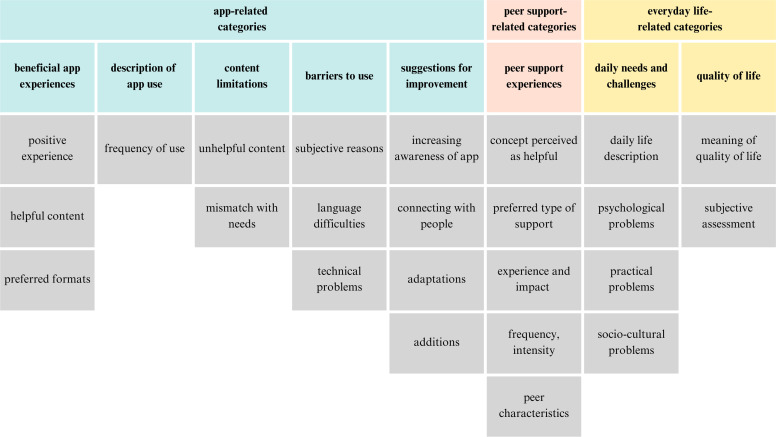
Eight main categories emerged from qualitative analysis with corresponding level 1 subcategories. A comprehensive overview including definitions, frequencies, and anchor examples of all detailed level 2 subcategories is provided in Appendix D.1.

Fig. 5 displays the main categories from the qualitative interviews (*N* = 41): beneficial app experiences, description of app use, content limitations and barriers, suggestions for improvement, peer support experiences, daily challenges and quality of life. A comprehensive overview including definitions, frequencies, and anchor examples is provided in Appendix D.1.

Most participants reported positive experiences with the Sui app, particularly regarding the provision of helpful, clear, and relevant information (58.5 %). A majority of respondents appreciated the general quality of the app (56.1 %), its ease of use (43.9 %), and the breadth and depth of content (36.6 %). Some highlighted well-being chapters and audio exercises, while others were more interested in information chapters. Video testimonials were often cited as motivating and relatable (43.9 %). Furthermore, participants expressed a format preference for videos (39.0 %) and audio exercises (36.6 %), with fewer favouring written formats (12.2 %).

The use of app varied: while some used it frequently, most accessed it as needed (26.8 %), depending on current life situations. Some of the respondents described a mismatch between the information provided and their real-life needs, mainly due to missing actionable steps (29.3 %) or individualised information (19.5 %). Others found the well-being chapters too superficial (9.8 %) or irrelevant to their needs (17.1 %). Further barriers included unclear purpose (24.4 %), limited personal time (7.3 %), technical issues (loading time: 12.2 %, login issues: 9.8 %), and language difficulties (12.2 %). Participants suggested clearer onboarding (e.g., an explanation video) and more direct links to local services. A strong need was expressed for daily support and personal contact, either through peers or professionals, to help navigate integration processes.

Peer support was well-received (19.5 %), and those who did not use it were largely enthusiastic about the concept (14.6 %). However, some participants did not fully understand (14.6 %) or failed to notice this feature at all (12.2 %). Interviews also identified daily practical challenges of the respondents such as language barriers (41.5 %), lack of information (41.5 %), occupational integration (29.3 %), and financial instability (26.8 %) to name but a few.

## 4 Discussion

This study evaluated the effectiveness and acceptability of the Sui app, a culturally adapted digital psychosocial service for Arabic-speaking refugees in Switzerland. While the app aimed to improve QOL and mental health outcomes, the findings showed no statistical improvements, except for two significant interaction effects at follow-up. These results must be viewed in light of the app’s design, study conduct, and user engagement. Qualitative interviews provided valuable insights into participants’ reception and suggestions to improvement.

At the 8-week post-assessment, no significant group-by-time interactions were found in the primary outcome domains. Satisfaction with the app was good, with a mean score of 2.81 out of 4. At the 16-week follow-up, a significant interaction in the psychological domain was found with a very small effect size between the two active groups, potentially driven by the baseline difference between the study groups in this domain. No significant interactions were identified for the secondary outcomes at post-assessment. At follow-up, stigma agreement (SSMIS-SF, Section 2) showed a significant interaction with a small effect size. However, the self-stigma measure demonstrated very low internal consistency (α = 0.37) in the current sample, likely reflecting difficulties in item comprehension and the use of an Arabic translation that had not been formally validated. This substantially limits the reliability and interpretability of this finding. Moreover, it is important to note that the control group was not included in the follow-up analyses, as they had gained access to the app in the meantime.

When interpreting the results, it is necessary to consider the app usage time across all available participant data (*M* = 13.1 min). The amount tripled if only the 95 verified users that successfully logged into the app were taken into account (*M* = 34.5 min). Interview participants identified barriers such as unclear app purpose, personal time constraints, technical issues, and missing tailored app guidance, all of which likely reduced engagement. The app’s flexible design, without a standardised or modular structure, likely contributed to lower engagement, but also presents an opportunity to explore valuable user data on non-linear platforms. This user autonomy approach is rare in the literature and provides important insights.

A review of digital approaches improving QOL reported highly variable studies regarding their intervention design, methods, targeted outcomes, often targeting populations with elevated psychopathological symptoms, such as depression (Nascimento et al., 2023). Unlike most preceding intervention studies, which typically employed a modular structure and focused on populations with predefined clinical cut-offs, our study targeted a broader, more heterogeneous population. This may have contributed to the lack of clarity of purpose and lower engagement. For instance, a linear app targeting depression in refugees (Carswell et al., 2018) reported higher engagement and improvements of psychological distress (Burchert et al., 2024) and depression (Cuijpers et al., 2022), but still faced challenges in achieving expected engagement. Another fully self-guided app with a modular structure, designed to reduce posttraumatic stress symptoms in Syrian refugees in Germany, reported an average usage time of 42.5 min over four weeks, however failed to demonstrate effectiveness (Röhr et al., 2021). In that study, approximately one-third of participants did not progress beyond the initial onboarding module. Similarly, in our study, around 30 % of participants did not successfully log into the app. These findings highlight the common challenge of ensuring smooth onboarding and sustained engagement in digital interventions (Baumel et al., 2019; Donkin et al., 2011).

While many interview participants appreciated the information provided, they described it as too general or difficult to apply in practice, particularly in areas like housing or legal procedures. These insights point to a critical implementation gap: the app successfully delivered relevant content but did not adequately support users in translating knowledge into action. This gap helps to contextualise the lack of measurable change in QOL, despite generally positive user satisfaction. The app’s content itself was well aligned with participants’ descriptions of their daily challenges and needs, such as legal uncertainty, language barriers, housing difficulties, and social isolation. This reflects the app’s context-sensitive design in line with core principles of MHPSS services (IFRC Reference Centre for Psychosocial Support 2020).

Surprisingly, the addition of weekly peer support did not lead to measurable improvements in QOL or symptom severity. While support through trained non-clinicians has been shown to improve mental health outcomes and engagement in other digital interventions (Leung et al., 2022; Musiat et al., 2022), no such benefits were observed in this study. The engagement with peer support was low, as fewer than half of the participants in the Sui+ group actively interacted with their assigned peer and no significant differences in usage time was observed between the active groups. Qualitative feedback suggested that participants either did not see or trust the peer messages, possibly due to deactivated notifications and standard text templates (impression of a bot). Nevertheless, participants who engaged more frequently with peer support reported highly positive experiences and valued the personal contact. The findings suggest that the potential of peer support was not fully realised. Moving forward, these qualitative results underline the need for more interactive and personalised app features, stronger onboarding and guidance, and more visible and responsive support elements to increase both usage and impact.

Strengths of this study include the mixed-methods approach and the high participation rate in qualitative interviews, which provided rich insights. Additionally, the inclusion of culturally sensitive content, which received minimal criticism further supports the relevance and acceptance of the app. Unlike many previous studies focusing exclusively on Syrian refugees, this study included Arabic-speaking refugees from diverse countries, broadening accessibility. Language concerns about content provided in the app were not related to participants’ national origin, but rather to differences in literacy and education levels. This finding adds to the growing body of research on contextual adaptation beyond traditional cultural adaptation.

According to a systematic review, the WHOQOL-BREF is suitable for detecting change regardless of the time elapsed between measurements (Skevington and Epton, 2018). Nonetheless, the potential for such changes may be limited by a ceiling effect, as the QOL scores in our sample were not substantially lower than those observed in general populations (Skevington et al., 2004). This likely restricted the ability to detect improvements and suggests that we might not have studied a representative sample. Including only refugees with lower QOL could have increased the likelihood of observing measurable change. However, the decision to include all refugees aligned with the app’s goal of supporting individuals across varying levels of need, particularly those underserved or with limited access to traditional mental health services.

The study presents several limitations that should be considered. First, the dropout of more than 30 % at post and follow-up assessments may have introduced a bias, potentially limiting the generalisability of the results. Second, the reliance solely on self-reported measures, without incorporating observer ratings, may have affected the objectivity of the data. Third, the study did not examine long-term effects, which are critical for understanding the sustained impact of the app. As highlighted by previous authors reviewing digital interventions, detecting meaningful changes in QOL might require long-term follow-up assessments (Hennemann et al., 2018). Fourth, the internal consistencies of the social relations domain of the WHOQOL-BREF and of the SSMIS-SF subscales were low, which restricts the interpretability of findings involving these outcomes. Fifth, qualitative findings were limited by potential loss of meaning in translation, and the results might not be generalisable, as the sample was self-selected.

Future research should seek to address these limitations and integrate the aforementioned suggestions to enhance user engagement and overall effectiveness. Given that the app was designed to be easily adaptable for other linguistic refugee populations, it would be valuable to test it with different groups to further explore user behaviour. Further analyses of our data should investigate which participants benefitted most and explore factors that could enhance the effectiveness of the app for those who experienced limited benefits.

Despite its limitations, this study marks a foundational step for digital psychosocial services that actively integrate the post-migration context by embedding local information within their mental health agenda. To our knowledge, there is no existing evidence of digital psychosocial services employing a similar concept. Moreover, the lack of evaluated digital interventions for refugees, especially smartphone-based, targeting improvement in QOL as a primary outcome emphasises the novelty and importance of this research. The well-accepted content, design and the holistic concept of the app provide a valuable foundation for further development and refinement, offering potential for broader application and impact in future digital MHPSS interventions.

## Funding

The authors acknowledge the financial support from the Humanitarian Foundation of the Swiss Red Cross. The funding did not influence the design of the study, the collection, management, analyses, or interpretation of the data, the writing of the manuscript, or the selection of the journal.

## Declaration of generative AI and AI-assisted technologies in the writing process

During the preparation of this work the author(s) used ChatGPT-4 in order to improve language and readability. After using this tool, the author(s) reviewed and edited the content as needed and take(s) full responsibility for the content of the publication.

## CRediT authorship contribution statement

**Rilana T Stoeckli:** Writing – original draft, Visualization, Supervision, Software, Project administration, Methodology, Investigation, Formal analysis, Data curation, Conceptualization. **Viktoria Zoellner:** Writing – review & editing, Supervision, Project administration, Investigation, Conceptualization. **Farhad Haji:** Writing – review & editing, Supervision, Investigation, Conceptualization. **Monia Aebersold:** Writing – review & editing, Supervision, Project administration, Funding acquisition, Conceptualization. **Sebastian Burchert:** Writing – review & editing, Software, Resources. **Jessica Wabiszczewicz:** Writing – review & editing, Software, Resources. **Christine Knaevelsrud:** Writing – review & editing, Software, Resources. **Eva Heim:** Writing – review & editing, Conceptualization. **Thomas Berger:** Writing – review & editing, Supervision, Resources, Conceptualization.

## Declaration of competing interest

The authors declare that they have no known competing financial interests or personal relationships that could have appeared to influence the work reported in this paper.

## Appendix Supplementary materials

Image, application 1
