# Supplementary material for: Psychosocial support for Arabic-speaking refugees residing in Switzerland (Sui app): A mixed-methods randomised controlled trial
Source: J Migr Health. 2025 Nov 27;12:100379. doi: 10.1016/j.jmh.2025.100379 (PMC12720314; doi:10.1016/j.jmh.2025.100379)
Supplement: Supplementary file 1 [file mmc1.docx]

**Appendices**

**Appendix A.1-Interview guide**

**Interview guide 1 (with / without peer support)**

Hello. I'm xx from the Sui app study team. We have an appointment for a telephone interview today, is that correct?

Thank you for agreeing to talk to us about the Sui app. You are helping us to improve the app and develop it further so that others can benefit from it too.

In the following, we will first talk about the app in general and then about more specific topics. You are welcome to say anything you can think of at any time and be completely open and honest - this is the only way we can improve our offering. If you would rather not answer a question or are unable to answer it, you can say so at any time. Your details will be anonymised and treated confidentially. It will not be possible to draw any conclusions about you as a person.

If you do not understand something, please ask at any time. I would like to record the interview so that I can focus as much as possible on what you have to say. After the interview has been processed, the recording will be permanently deleted. Do you agree to this?

The recording is not running at the moment. I will let you know as soon as I start the recording. Now I'd like to say something about the structure of the interview. The interview will take about an hour and will consist of 4 parts. Firstly, there will be some general questions about the app. Then there are questions about well-being. The third part is about the support of a peer companions. The last part is about quality of life.

Do you have any questions so far?

If not, we can start the interview.

Is it okay for you if I start the audio recording now?

*START AUDIO RECORDING*

**General (UX UI/content): 10 min**

Introduction: First, I will ask you some general questions about the app.

1^st^ priority questions:

1. How often have you used the app? Why approximately so often?
2. What did you particularly like about the app?
   1. Help: Look and feel, stability, functions, speed, navigation, content
3. What did you not like about the app?
   1. If not mentioned: Did you find anything impractical or confusing?
4. Apart from the content: What did you think of the technical aspect of the app?
   1. Help: e.g. the colours, handling/navigation in the app?
5. How do you think the app can help people who are new to Switzerland?
6. Did the app help you to find your way around Switzerland better?
   1. If yes: How did it help you?
   2. If no: Why not?
7. How will you use the app in the future?

2^nd^ priority questions:

1. Which topics are most important for Arabic-speaking refugees in everyday life?
2. Can you think of any reasons why Arabic-speaking refugees do not use the app?

3^rd^ priority questions:

1. Think about what is important to you in everyday life. Was any of this missing in the app? e.g. content, functions, exercises, information

**Psychological topics: 15 min**

Introduction: One part of the app deals with the topic of “well-being”. These chapters are dark green and can be found at the bottom of the home screen. This part was mainly about mental health and how to deal with difficult life situations. The next questions are about this part.

1^st^ priority questions:

1. Which dark green chapters on well-being did you use? (stress, sleep, daytime resources, regulating emotions, chronic pain, audio exercises)
   1. Which chapters and exercises did you like? Why?
   2. Which ones less? Why?
2. Do you now think differently about the topic of well-being (e.g. stress, sleep, regulating emotions, chronic pain)?
3. Has the app influenced you to take more care of your own health in general?
   1. If yes: What examples and/or situations can you think of?

2^nd^ priority questions:

1. How important are the dark green chapters on well-being (stress, sleep, resources during the day, regulating emotions, chronic pain) for you personally?

**Participants without peer support: 15 min**

Introduction: The SRC would like to expand the app in future so that users of the service can receive additional support from trained Arabic-speaking peers via chat.

1^st^ priority questions:

1. Would it have helped you if you had received additional personal support in the chat?
   1. If yes: what content and challenges?
2. What would optimal support look like for you?
3. Who should the counsellor be?
   1. A professional? A trained person with the same mother tongue? Someone else?
4. How important is it for you that the accompanying person has experienced something similar to you?

2^nd^ priority questions:

1. Would an accompanying person have helped you to use the app more?
2. Why and how would it have helped?

**User with peer support: 15 min**

Introduction: Over the last 8 weeks, you have had a companion who has sent you messages every week. I will ask you the next questions about this companion.

1^st^ priority questions:

1. Did the counselling help you?
   1. If yes: What has helped you? If nothing: The suggestions, feedback or motivation to use the app?
   2. If no: why not? What did not help you with the counselling?
2. Would you like to have a counsellor beyond the study?
   1. If yes/if nothing at all: what format, what intervals between messages, for a certain period of time, on call?
   2. If yes: Assuming you continue to have a support person: What questions and concerns would you write to the support person with?
3. How could the support be improved?
4. In your opinion, does it make a difference if you are accompanied while using the app?
   1. If yes/no: Why?

​​2^nd^ priority questions:

1. Did the support help you to use the app more?
   1. If yes: How did the accompanying person help you to use the app more?
   2. If no: what would you have needed from the peer to make you use the app more?
2. How did you use the peer support during the 8 weeks?
3. How important is it to you that the peer support experienced something similar to you?

3^rd^ priority questions:

1. What did you like about the messages?
   1. What did you find less good about the messages?
2. Can you remember a message that was particularly helpful to you?
3. What was your level of trust in the counsellor?

**Quality of life: 15 min**

Introduction: Now we come to the last part, the quality of life.

In this study, we investigated whether the quality of life of people who used the app improved. To do this, we asked you standard questions before and after using the app. Now, however, we would like to know from you specifically what you consider to be a good quality of life.

1^st^ priority questions:

1. What does it mean to you to have a good quality of life?
   1. If there is enough time: What do you think the everyday life of a person with a good quality of life looks like?
2. How would you rate your quality of life?
3. What is good for you in your life?
4. What makes life rather difficult for you?
5. What do you think would help to improve your quality of life - and that of your family or friends?
6. What would a ‘perfect’ app have to look like if it were to generally improve your quality of life or support you in case of difficulties?

2^nd^ priority questions:

1. what is particularly important to you in life?
2. how satisfied are you with the things that are particularly important to you in life?

3^rd^ priority questions:

1. Have you noticed any changes in your quality of life compared to before using the app?
   1. If yes: What changes?
   2. If yes: What content of the app do you think has contributed to improving your quality of life?
2. What content did NOT contribute to this improvement?
3. What would you change about the app to improve your quality of life?

**Closing question: 1 min**

Introduction: We are slowly approaching the end of the interview. I have one final question for you.

1. Would you recommend the app to a friend?

If yes: in what words? If no: why not?

**FAQ/feedback: 5 min**

We have now reached the end of the interview. Thank you very much for answering the questions! Do you have any further feedback or questions about the interview?

**Conclusion**

I would like to thank you once again for taking the time for the interview. You will receive another link to the final questionnaire in 8 weeks. After that, you will also receive a personalised link and a code by text message with which you can download your Migros voucher - that's CHF 20 per questionnaire, i.e. CHF 60 in total if you have completed all three questionnaires.

Do you have any further questions?

Then thank you again; I wish you all the best!

**Appendix A.2-Interview guide**

**Interview guide 2**

Hello, I am cc from the Sui app study team at the University of Bern. We are scheduled for a phone interview today, is that correct?

Thank you for agreeing to talk to us about the Sui App. By doing so, you are helping us improve and develop the app, so that others can benefit from it as well.

We will first discuss the app in general, then we would like to ask a few questions about your current situation and where you may generally need support in your daily life.

You are welcome to share anything that comes to mind and be completely open and honest – only in this way can we improve our offering. If there is a question you would prefer not to answer, or if you are unable to answer it, you can let me know at any time. Your responses will be anonymized and treated confidentially. No conclusions can be drawn about you as an individual.

If there is anything you don't understand, feel free to ask at any time. To focus best on your responses, I would like to record this conversation. After the interview is processed, the recording will be permanently deleted. Do you agree to this?

The recording has not started yet. I will inform you once I begin the recording.

The interview will last about half an hour.

Do you have any questions so far?

If not, we can start the interview.

Is it okay for you if I start the audio recording now?

*START AUDIO RECORDING*

**Introduction: 2 minutes**

1. How did you come across the Sui study?

**Part 1: Satisfaction with the app: 10 minutes**

Introduction: First, I will ask you a few questions about the Sui app. You have already filled out a long questionnaire, in which you also answered questions about the Sui app. During your participation in the study, we were also able to see how often and how long you used the Sui app each week. These are already interesting and important pieces of information for us, but we would like to understand a bit more precisely how it was for you.

1. Would you like to share?

Questions based on user behaviour or questionnaire output:

| **Login** | **Questions** |
| --- | --- |
| Logged in only once | You logged into the app once. Can you describe what that time was like for you? What did you like about the app, and what did you like less? What could have helped you use the app more? |
| Logged in repeatedly, but briefly | You logged into the app repeatedly, but quickly closed it again. Can you describe why that might have been the case? |
| Logged in repeatedly, but for a bit longer | You logged into the app repeatedly. Can you describe what that time was like for you? What did you like about the app, and what did you like less? |
| Logged in frequently and for long periods | You logged into the app frequently and were active in the app for longer periods. Can you describe what that time was like for you? What did you like about the app, and what did you like less? |

| **Login without peer support** | **Questions** |
| --- | --- |
| No interaction with peer | You logged into the app once but did not interact with your companion. Can you describe what that time was like for you? What did you like about the app, and what did you like less? Why did you not interact with your companion? |
| Little interaction with Peer | You logged into the app once and interacted with your companion at the beginning. Can you describe what that time was like for you? What did you like about the app, and what did you like less? |
| Much interaction with peer | You logged into the app repeatedly and also interacted with your companion. Can you describe what that time was like for you? What did you like about the app, and what did you like less? Did you continue using the app after your companion said goodbye? |
| A lot of interaction with peer | - |

| **Satisfaction with app** | **Questions** |
| --- | --- |
| Seems very dissatisfied | - |
| Seems somewhat dissatisfied | You answered in the questionnaire that you did not like the app and were rather dissatisfied with it. The app did not meet your needs. Can you explain that a bit more? What would need to be different in order to better meet your needs? |
| Seems satisfied | You answered in the questionnaire that you liked the app and were mostly satisfied. However, it met only a few of your needs. Can you explain that a bit more?  You answered in the questionnaire that you were mostly satisfied with the app, but it met only a few of your needs, and you are unlikely to use it again. Can you explain that in more detail? |
| Seems very satisfied | You answered in the questionnaire that you liked the app and were very satisfied, and the app met most of your needs. Can you explain that a bit more? |

**Follow-up questions:**

- Can you describe that in more detail? Can you explain what you mean by that?
- Which formats (videos, texts, etc.) in the app stood out to you or did you find particularly helpful?
- What could help in understanding the app better (e.g., an introductory video)? Was the app clear/easy to use?
- What could help in making the app be used more regularly/often?
- When logging in with a peer: What would make the interaction with the peer easier? (e.g., setting specific goals with the peer, etc.)
- Do you have other apps on your phone that you like to use?
- Do you have apps on your phone for your health?
- In which situations would you use the app again?

**Part 2: Needs in Life / External Factors**

1. What does your daily life look like at the moment?
2. What occupies you most in your life right now? What is taking up the most time in your life right now?
3. Which needs are you currently dissatisfied with in your life?
4. Where do you need support in your everyday life? a. What kind of support would be helpful/necessary?
5. How do you usually get information that you need for your life in Switzerland or for this particular topic or need?

**Follow-up questions:**

- In the Sui app, there is a section on "xxx" – have you seen that? Could that help?
- Would it even be possible to help you in this area/these areas with an app? If yes, what could that look like?
- What would you specifically want from an app that should support you in your daily life?
- Which topics should be present in such an app?
- What would help in continuing to use the Sui app? / What would need to be different in the Sui app for you to continue using it?

**Separate part: Technical Difficulties**

1. If never logged in: Why didn’t you use the app? Were there technical difficulties? What phone brand do you have? We are constantly improving the app, and sometimes issues depend on the phone brand.
   1. Loading times clarification: Yes, that’s true. The app sometimes takes a bit longer. How long did it take to load on your phone? Could it be related to your internet connection? What phone brand do you have? We are constantly improving the app, and sometimes issues depend on the phone brand.

**Information for the Study**

- If not yet completed: Please fill out the third questionnaire.
- If voucher already received: Please download the voucher by clicking on the link in the SMS and entering the code.
- If voucher not yet received: You will receive your voucher in the next few days. Please download it by clicking on the link in the SMS and entering the code.

**Appendix B.1-Baseline Outcomes**

Supplementary Table 1. Baseline comparisons of primary and secondary outcomes between study groups.

| **Measure** | **Sui+ (*n*=68)** | **Sui (*n*=68)** | **Waitlist (*n*=34)** | **Statistic** |
| --- | --- | --- | --- | --- |
| Quality of life, 4-20 *M* (*SD*) |  |  |  |  |
| Physical | 13.91 (2.67) | 14.75 (2.52) | 14.29 (2.88) | Kruskal-Wallis *χ^2^* (2) = 4.182 *p* = 0.124 |
| Psychological | 13.37 (2.60) | 14.72 (2.47) | 14.22 (2.36) | Kruskal-Wallis *χ^2^* (2) = 8.76 ***p* = 0.013*** |
| Social Relations | 13.42 (3.63) | 14.81 (2.68) | 14.27 (3.78) | Kruskal-Wallis *χ^2^* (2) = 3.737 *p* = 0.154 |
| Environment | 12.90 (2.65) | 12.40 (2.95) | 12.63 (3.18) | Kruskal-Wallis *χ^2^* (2) = 1.216 *p* = 0.544 |
| Depressive symptoms *M* (*SD*) | 8.91 (5.53) | 7.99 (5.16) | 6.56 (4.53) | Kruskal-Wallis *χ^2^* (2) = 5.038 *p* = 0.081 |
| Posttraumatic stress disorder symptoms *M* (*SD*) | 12.16 (7.98) | 10.31 (7.63) | 9.53 (7.99) | Kruskal-Wallis *χ^2^* (2) = 3.707 *p* = 0.157 |
| Anxiety symptoms *M* (*SD*) | 6.72 (4.80) | 5.22 (4.39) | 4.88 (4.00) | Kruskal-Wallis *χ^2^* (2) = 5.400 *p* = 0.067 |
| Somatic symptoms *M* (*SD*) | 8.56 (5.03) | 7.31 (5.49) | 7.04 (5.04) | Kruskal-Wallis *χ^2^* (2) = 2.653 *p* = 0.265 |
| Stigma awareness *M* (*SD*) | 3.94 (1.70) | 3.54 (1.71) | 3.83 (1.57) | Kruskal-Wallis *χ^2^* (2) = 1.823 *p* = 0.402 |
| Stigma agreement *M* (*SD*) | 3.72 (1.65) | 3.21 (1.81) | 3.53 (1.64) | Kruskal-Wallis *χ^2^* (2) = 3.718 *p* = 0.156 |
| Post-migration living difficulties *M* (*SD*) | 30.10 (12.54) | 28.10 (13.58) | 26.21 (11.96) | Kruskal-Wallis *χ^2^* (2) = 2.826 *p* = 0.243 |

**Appendix B.2-Internal Consistency**

*Supplementary Table 2. Internal consistency (Cronbach’s alpha) for all questionnaires at baseline.*

| **Measure** | **No. of items** | **Cronbach’s alpha** |
| --- | --- | --- |
| WHOQOL-BREF , Quality of life, 4-20 | 24 | .89 |
| Physical | 7 | .76 |
| Psychological | 6 | .74 |
| Social Relations | 3 | .61 |
| Environment | 8 | .83 |
| PHQ-9, Depressive symptoms | 9 | .86 |
| PCL-5, Posttraumatic stress disorder symptoms | 8 | .92 |
| GAD-7, Anxiety symptoms | 7 | .89 |
| PHQ-15, Somatic symptoms | 15 | .85 |
| SSMIS-SF, Self-Stigma | 10 | .57 |
| Section 1, Stigma awareness | 5 | .24 |
| Section 2, Stigma agreement | 5 | .37 |
| PMLD-CL, Post-migration living difficulties | 17 | .84 |

**Appendix C.1-Complete Outcome Table**

Supplementary Table 3. Observed and estimated means at all timepoints, and within- and between group effect sizes (ITT sample) at all timepoints.

| **Measure** | **Study group** | **Baseline** | | **Post (observed)** | | | **Post (estimated)** | | **Follow-up (observed)** | | **Follow-up (estimated)** | | **Baseline-post within-group effect sizes (estimated post means)** | **Group-by-time interaction (baseline, post)** | **Between-group effect sizes at post (estimated means)** | **Baseline-follow-up within-group effect sizes (estimated post means)** | **Group-by-time interaction (baseline, post, follow-up)** | **Between-group effect sizes at follow-up (estimated means)** |
| --- | --- | --- | --- | --- | --- | --- | --- | --- | --- | --- | --- | --- | --- | --- | --- | --- | --- | --- |
|  |  | **Mean (*SD*)** | ***n*** | **Mean (*SD*)** | ***n*** | **Mean (*SE*)** | | ***n*** | **Mean (*SD*)** | ***n*** | **Mean (*SE*)** | ***n*** | **Cohen’s *d* [95% CI]** | **F and *df*** | **Cohen’s *d* [95% CI]** | **Cohen’s *d* [95% CI]** | **F and *df*** | **Cohen’s *d* [95% CI]** |
| ↑ WHOQOL-BREF mean, 4-20 scale range  Physical Domain | Sui+ | 13.92 (2.67) | 68 | 13.86 (3.07) | 47 | 13.79 (0.37) | | 68 | 13.84 (2.85) | 44 | 13.93 (0.38) | 68 | 0.01 [-0.33; 0.34] | *F* _(2, 128.27)_ = 0.26  *p* = .77 | Sui+ vs. WL: -0.17 [-0.58; 0.24] | -0.00 [-0.34; 0.33] | *F* _(2, 190.05)_ = 0.98  *p* = .38 |  |
|  | Sui | 14.75 (2.52) | 68 | 14.10 (3.01) | 42 | 14.741 (0.39) | | 68 | 13.88 (2.91) | 46 | 14.16 (0.37) | 68 | 0.02 [-0.31; 0.36] |  | Sui+ vs. Sui: -0.20 [-0.54; 0.14] | 0.04 [-0.30; 0.38] |  | Sui+ vs. Sui: -0.07 [-0.41; 0.26] |
|  | WL | 14.29 (2.88) | 34 | 14.46 (3.07) | 27 | 14.31 (0.51) | | 34 | 14.78 (2.93) | 22 | --- | 34 | -0.00 [-0.48; 0.47] |  | Sui vs. WL: 0.03 [-0.38; 0.44] | --- |  |  |
| ↑ WHOQOL-BREF mean, 4-20 scale range  Psychological Domain | Sui+ | 13.37 (2.60) | 68 | 13.18 (2.96) | 47 | 13.17 (0.37) | | 68 | 13.44 (2.40) | 44 | 13.61 (0.37) | 68 | 0.01 [-0.32; 0.35] | *F* _(2, 134.67)_ = 1.85  *p* = .16 | Sui+ vs. WL: -0.29 [-0.70; 0.12] | -0.04 [-0.37; 0.30] | *F* _(2, 193.47)_ = 5.06  ***p* = .01*** |  |
|  | Sui | 14.72 (2.47) | 68 | 13.41 (3.25) | 42 | 13.66 (0.38) | | 68 | 13.39 (2.92) | 46 | 13.53 (0.37) | 68 | 0.07 [-0.26; 0.41] |  | Sui+ vs. Sui: -0.16 [-0.50; 0.18] | 0.08 [-0.25; 0.41] |  | Sui+ vs. Sui: 0.02 [-0.31; 0.36] |
|  | WL | 14.22 (2.36) | 34 | 13.93 (2.74) | 27 | 14.03 (0.50) | | 34 | 14.45 (2.54) | 22 | --- | 34 | 0.02 [-0.46; 0.49] |  | Sui vs. WL: -0.12 [-0.53; 0.29] | --- |  | --- |
| ↑ WHOQOL-BREF mean, 4-20 scale range  Social Relations Domain | Sui+ | 13.42 (3.63) | 68 | 13.11 (4.00) | 47 | 13.24 (0.47) | | 68 | 13.18 (3.82) | 44 | 13.39 (0.47) | 68 | 0.01 [-0.33; 0.34] | *F* _(2, 125.92)_ = 0.86  *p* = .42 | Sui+ vs. WL: -0.18 [-0.59; 0.24] | 0.00 [-0.33; 0.34] | *F* _(2, 191.46)_ = 0.92  *p* = .40 | --- |
|  | Sui | 14.81 (2.68) | 68 | 13.63 (3.08) | 42 | 13.86 (0.49) | | 68 | 13.80 (3.01) | 46 | 14.13 (0.46) | 68 | 0.06 [-0.28; 0.40] |  | Sui+ vs. Sui: -0.16 [-0.50; 0.18] | 0.04 [-0.29; 0.38] |  | Sui+ vs. Sui: -0.20 [-0.53; 0.14] |
|  | WL | 14.27 (3.78) | 34 | 14.02 (3.61) | 27 | 13.90 (0.63) | | 34 | 14.42 (3.51) | 22 | --- | 34 | 0.02 [-0.45; 0.50] |  | Sui vs. WL: -0.01 [-0.42; 0.40] | --- |  | --- |
| ↑ WHOQOL-BREF mean, 4-20 scale range  Environment Domain | Sui+ | 12.40 (2.95) | 68 | 12.68 (2.77) | 47 | 12.60 (0.37) | | 68 | 12.96 (2.52) | 44 | 12.97 (0.37) | 68 | -0.01 [-0.35; 0.32] | *F* _(2, 120.67)_ = 0.56  *p* = .57 | Sui+ vs. WL: -0.05 [-0.46; 0.36] | -0.03 [-0.37; 0.30] | *F* _(2, 184.69)_ = 1.61  *p* = .20 | --- |
|  | Sui | 12.90 (2.65) | 68 | 12.33 (2.54) | 42 | 12.69 (0.39) | | 68 | 12.48 (2.67) | 46 | 12.75 (0.36) | 68 | 0.01 [-0.32; 0.35] |  | Sui+ vs. Sui: -0.03 [-0.37; 0.31] | 0.01 [-0.33; 0.35] |  | Sui+ vs. Sui: 0.07 [-0.26; 0.41] |
|  | WL | 12.63 (3.18) | 34 | 13.00 (3.05) | 27 | 12.75 (0.51) | | 34 | 13.27 (2.09) | 22 | --- | 34 | -0.01 [-0.48; 0.47] |  | Sui vs. WL: -0.02 [-0.43; 0.39] | --- |  | --- |
| ↓ PHQ-9 sum,  0-27 range  Depressive symptoms | Sui+ | 8.91 (5.53) | 68 | 9.04 (6.82) | 46 | 9.34 (0.77) | | 68 | 9.45 (5.63) | 42 | 11.62 (1.13) | 68 | -0.01 [-0.34; 0.33] | *F* _(2, 125.79)_ = 0.68  *p* = .51 | Sui+ vs. WL: 0.39 [-0.02; 0.81] | -0.01 [-0.34; 0.33] | *F* _(2, 185.96)_ = 1.26  *p* = .29 | --- |
|  | Sui | 7.99 (5.16) | 68 | 9.74 (6.68) | 42 | 9.43 (0.80) | | 68 | 7.76 (4.64) | 45 | 10.40 (1.11) | 68 | -0.03 [-0.37; 0.31] |  | Sui+ vs. Sui: -0.01 [-0.35; 0.32] | 0.01 [-0.33; 0.34] |  | Sui+ vs. Sui: 0.25 [-0.09; 0.59] |
|  | WL | 6.56 (4.53) | 34 | 6.96 (5.68) | 26 | 7.08 (1.05) | | 34 | 6.86 (6.16) | 22 | --- | 34 | -0.02 [-0.49; 0.46] |  | Sui vs. WL: 0.37 [-0.05; 0.78] | --- |  | --- |
| ↓ PCL-5 sum,  0-32 range  8-item version  Posttraumatic stress disorder symptoms | Sui+ | 12.16 (7.98) | 68 | 11.67 (9.00) | 46 | 12.39 (1.10) | | 68 | 11.83 (8.31) | 42 | 11.62 (1.13) | 68 | -0.00 [-0.34; 0.33] | *F* _(2, 121.22)_ = 1.39  *p* = .25 | Sui+ vs. WL: 0.50 [0.08; 0.91] | 0.01 [-0.32; 0.35] | *F* _(2, 183.92)_ = 0.12  *p* = .89 | --- |
|  | Sui | 10.31 (7.63) | 68 | 11.64 (9.19) | 42 | 11.17 (1.12) | | 68 | 10.80 (7.98) | 45 | 10.40 (1.11) | 68 | -0.02 [-0.36; 0.32] |  | Sui+ vs. Sui: 0.13 [-0.20; 0.47] | -0.00 [-0.34; 0.33] |  | Sui+ vs. Sui: 0.13 [-0.20; 0.47] |
|  | WL | 9.53 (7.99) | 34 | 7.38 (7.34) | 26 | 8.00 (1.50) | | 34 | 7.73 (7.67) | 22 | --- | 34 | 0.05 [-0.43; 0.52] |  | Sui vs. WL: 0.35 [-0.06; 0.77] | --- |  | --- |
| ↓ GAD-7 sum,  0-21 range  Anxiety symptoms | Sui+ | 6.72 (4.80) | 68 | 7.02 (5.89) | 46 | 7.19 (0.68) | | 68 | 7.12 (5.56) | 42 | 6.83 (0.71) | 68 | -0.02 [-0.35; 0.32] | *F* _(2, 132.18)_ = 2.34  *p* = .10 | Sui+ vs. WL: 0.54 [0.12; 0.96] | -0.00 [-0.34; 0.33] | *F* _(2, 191.41)_ = 0.61  *p* = .55 | --- |
|  | Sui | 5.22 (4.39) | 68 | 7.07 (6.04) | 42 | 6.79 (0.70) | | 68 | 5.78 (4.94) | 45 | 5.41 (0.70) | 68 | -0.06 [-0.40; 0.28] |  | Sui+ vs. Sui: 0.07 [-0.27; 0.41] | -0.01 [-0.34; 0.33] |  | Sui+ vs. Sui: 0.24 [-0.09; 0.58] |
|  | WL | 4.88 (4.01) | 34 | 4.08 (4.63) | 26 | 4.24 (0.92) | | 34 | 4.32 (4.11) | 22 | --- | 34 | 0.04 [-0.37; 0.46] |  | Sui vs. WL: 0.45 [0.04; 0.87] | --- |  | --- |
| ↓ PHQ-15 sum,  0-30 range  Somatic symptoms | Sui+ | 8.55 (5.56) | 68 | 9.07 (6.80) | 46 | 9.03 (0.75) | | 68 | 10.00 (6.38) | 42 | 9.65 (0.82) | 68 | -0.01 [-0.35; 0.32] | *F* _(2, 123.97)_ = 1.74  *p* = .18 | Sui+ vs. WL: 0.41 [-0.00; 0.83] | -0.03 [-0.37; 0.30] | *F* _(2, 188.32)_ = 1.03  *p* = .36 | --- |
|  | Sui | 6.90 (5.19) | 68 | 9.01 (6.54) | 42 | 8.46 (0.77) | | 68 | 8.78 (6.40) | 45 | 8.31 (0.80) | 68 | -0.05 [-0.39; 0.28] |  | Sui+ vs. Sui: 0.09 [-0.24; 0.43] | -0.05 [-0.38; 0.29] |  | Sui+ vs. Sui: 0.20 [-0.14; 0.54] |
|  | WL | 6.65 (4.85) | 34 | 5.80 (5.73) | 26 | 6.53 (1.03) | | 34 | 5.98 (4.26) | 22 | --- | 34 | 0.01 [-0.47; 0.48] |  | Sui vs. WL: 0.31 [-0.10: 0.72] | --- |  | --- |
| ↓ SSMIS-SF  1-9 mean range  Section 1: Stigma awareness | Sui+ | 3.94 (1.70) | 68 | 3.60 (2.15) | 46 | 3.52 (0.26) | | 68 | 3.63 (1.47) | 41 | 3.61 (0.27) | 68 | 0.04 [-0.29; 0.38] | *F* _(2, 138.49)_ = 0.63  *p* = .54 | Sui+ vs. WL: 0.03 [-0.38; 0.45] | 0.03 [-0.30; 0.37] | *F* _(2, 199.99)_ = 0.86  *p* = .42 | --- |
|  | Sui | 3.54 (1.71) | 68 | 3.67 (1.78) | 42 | 3.53 (0.27) | | 68 | 3.70 (1.86) | 46 | 3.57 (0.25) | 68 | 0.00 [-0.33; 0.34] |  | Sui+ vs. Sui: -0.00 [-0.34; 0.33] | -0.00 [-0.34; 0.33] |  | Sui+ vs. Sui: 0.02 [-0.32; 0.35] |
|  | WL | 3.83 (1.57) | 33 | 3.44 (1.99) | 27 | 3.45 (0.34) | | 34 | 3.51 (1.95) | 22 | --- | 34 | 0.06 [-0.42; 0.53] |  | Sui vs. WL: 0.04 [-0.37; 0.45] | --- |  | --- |
| ↓ SSMIS-SF  1-9 mean range  Section 2: Stigma agreement | Sui+ | 3.72 (1.65) | 68 | 3.00 (1.75) | 46 | 3.00 (0.24) | | 68 | 3.47 (1.63) | 42 | 3.45 (0.25) | 68 | 0.07 [-0.26; 0.41] | *F* _(2, 135.13)_ = 2.61  *p* = .08 | Sui+ vs. WL: -0.17 [-0.58; 0.25] | 0.03 [-0.31; 0.36] | *F* _(2, 192.9)_ = 3.50  ***p* = .03*** | --- |
|  | Sui | 3.21 (1.81) | 68 | 3.44 (1.63) | 42 | 3.29 (0.25) | | 68 | 3.38 (1.84) | 45 | 3.21 (0.24) | 68 | -0.01 [-0.34; 0.33] |  | Sui+ vs. Sui: -0.14 [-0.48; 0.19] | 0.00 [-0.34; 0.34] |  | Sui+ vs. Sui: 0.12 [-0.21; 0.46] |
|  | WL | 3.53 (1.64) | 34 | 3.33 (1.87) | 27 | 3.32 (0.32) | | 34 | 3.33 (1.93) | 22 | --- | 34 | 0.03 [-0.45; 0.51] |  | Sui vs. WL: -0.02 [-0.43; 0.40] | --- |  | --- |
| ↓ PMLD-CL  0-68 range  Post-migration living difficulties | Sui+ | 30.09 (12.54) | 68 | 28.46 (13.51) | 46 | 29.08 (1.81) | | 68 | 29.23 (13.05) | 43 | 28.73 (1.79) | 68 | 0.01 [-0.32; 0.35] | *F* _(2, 127.30)_ = 1.02  *p* = .36 | Sui+ vs. WL: 0.46 [0.04; 0.88] | 0.02 [-0.32; 0.35] | *F* _(2, 186.99)_ = 0.09  *p* = .91 | --- |
|  | Sui | 28.10 (13.58) | 68 | 28.40 (14.33) | 42 | 27.90 (1.86) | | 68 | 26.63 (13.12) | 46 | 26.39 (1.76) | 68 | 0.00 [-0.33; 0.34] |  | Sui+ vs. Sui: 0.08 [-0.26; 0.42] | 0.02 [-0.31; 0.36] |  | Sui+ vs. Sui: 0.16 [-0.18; 0.50] |
|  | WL | 26.21 (11.96) | 34 | 22.52 (15.06) | 27 | 22.41 (2.43) | | 34 | 26.68 (16.27) | 22 | --- | 34 | 0.08 [-0.40; 0.55] |  | Sui vs. WL: 0.37 [-0.04; 0.79] | --- |  | --- |

Notes. WHOQOL-BREF = World Health Questionnaire Quality of Life Short Version, PHQ-9 = Depression Module of Patient Health Questionnaire, PCL-5 = Posttraumatic Stress Disorder Checklist for DSM-5, GAD-7 = Generalized Anxiety Disorder Screening, PHQ-15 = Somatic Module of Patient Health Questionnaire, SSMIS-SF = Self-Stigma of Mental Illness Scale Short Form, PMLD-CL = Post-Migration Living Difficulties Checklist. The symbol ↑ indicates that higher values mean better health, symbol ↓ indicates that lower values mean better health

**Appendix D.1-Qualitative Results**

Supplementary Table 4. Categories, subcategories, definitions, and anchor examples of the qualitative analysis (N = 41)

| **Main**  **categories** | **Subcategories level 1** | **Subcategories level 2** | **Definition** | ***n* (%)** | **Anchor example** |
| --- | --- | --- | --- | --- | --- |
| **Beneficial app experiences** |  |  |  |  |  |
|  | Positive experience |  |  |  |  |
|  |  | Helpful information | This subcategory reflects users' perceptions of receiving valuable, practical, or useful information from the Sui app. Helpful information typically refers to content that aids in understanding processes, navigating daily life, or solving specific problems, especially for individuals who are new to Switzerland. | 24 (58.5) | "It was a very helpful and good experience with the Sui app. There were many helpful pieces of information and useful tips that you can use. For example, when it comes to searching for housing, how you can search, where to search, and especially how to interact with landlords or during viewings, what to pay attention to." |
|  |  | The app is generally good | Participants who shared positive experiences with the Sui app, highlighting its helpfulness, structure, and user-friendly features. They appreciated the variety of information it provided, its ease of use, and its relevance to daily life in Switzerland. | 23 (56.1) | "It’s a really great app, very helpful for daily life. There’s a lot of useful information about things you don’t know and that can cause stress. You can look it up in the app, and it’s very useful." |
|  |  | Easy/clear to use | Refers to the ease and clarity with which a user can the app. It emphasizes intuitive navigation, simple design, and clear information that does not require additional explanation or support. | 18 (43.9) | "Everything was divided by title, and it was clear" |
|  |  | High informational content | This subcategory captures instances where participants expressed positive feedback about the abundance of information provided by the Sui app. | 15 (36.6) | The app really has a lot of information, very broad and detailed information, there's nothing missing." |
|  |  | Nothing negative about the app | This subcategory reflects the overwhelming positive feedback from participants, indicating that there was nothing they disliked or found problematic in the app. | 14 (34.1) | "The app, I can't say anything, it's almost perfect. What it offers is excellent." |
|  |  | Well-being chapters generally helpful | This subcategory reflects users' positive experiences with the app's well-being chapters, particularly topics related to mental health, stress management, and general well-being, which users found useful in improving their overall health and coping strategies. | 13 (31.7) | "It was a good experience. I kept looking at the sections about well-being, especially stress, or exercises, because at the asylum centre, it can get really stressful. The information was helpful, especially the ones that you could apply." |
|  |  | New information for target group | Participants appreciate learning about topics they were not aware of before, such as legal rights, work regulations, or integration tips in Switzerland. The app helps fill gaps in their knowledge, making it a useful tool for newcomers who are trying to navigate their new environment. | 7 (17.1) | "There were many helpful pieces of information, many new things that I had not known before, like topics on housing, asylum, and my legal status here in Switzerland. The app provided detailed, clear explanations on how to search for housing and how the system works here." |
|  |  | Improved well-being through the app | This subcategory reflects how users report improvements in their overall well-being, such as reduced stress, better mental health, and enhanced coping strategies, as a result of using the app. | 6 (14.6) | "It has helped me a bit because there was a lot of information and helpful tips. The way they shared the stories of people who had gone through similar experiences was really helpful. It brought me new ideas and made me think: if this person handled this situation this way, what can I do in a similar situation?" |
|  |  | App available in Arabic | This subcategory refers to users' positive experiences when the app offers content in Arabic, making it easier for Arabic-speaking individuals to access and understand important information related to their integration in Switzerland. | 4 (9.8) | "The best part of the app for me was that it was available in Arabic. Since I speak Arabic well, this made everything much easier to understand, and it was the most important feature for me." |
|  |  | Protection from misinformation | This subcategory refers to the user's experience of receiving accurate, reliable information through the app, protecting them from false or misleading content. | 3 (7.3) | "People who come to Switzerland often don't have information or get wrong information. [...] Not everyone can use Google or contact lawyers. [...] How long do I have to wait for XX? All of this is provided in the app in the native language." |
|  | Helpful content |  |  |  |  |
|  |  | Testimonials | Participants stating that personal accounts or stories shared by others, whether in video or written form, are helpful in providing insights into experiences, challenges, and solutions, offering valuable guidance or motivation for navigating similar situations. | 18 (43.9) | "What I liked the most were the videos of people who shared their experiences. For instance, one person mentioned that if you are highly qualified or looking for a job in Switzerland, you should ask people who have been here for a while. This is something I want to implement now, to seek out people who have been here longer so I can ask them for advice." |
|  |  | Asylum procedure / residence status | This subcategory refers to the user's satisfaction with the information provided by the app about the asylum process, residence status, rights, and duties related to different types of permits (e.g., F or B permits). Users found this content particularly useful when navigating their legal status in Switzerland and understanding the conditions tied to their asylum process. | 13 (31.7) | "The app was very useful, especially for understanding my residence status and the rights and duties tied to my F permit. It helped me learn what I can do and what my rights are, such as what to do when transitioning from one permit to another. This part of the app was especially helpful to me." |
|  |  | Work / education | This subcategory refers to the positive feedback regarding the helpfulness of information found in the Sui app concerning employment, educational opportunities, and related rights and responsibilities. Users appreciate the guidance on job applications, work rights, educational paths, and the process of obtaining qualifications in Switzerland. | 13 (31.7) | "The chapter on work and education was especially helpful, very, very helpful. Especially to find out what you can do and what opportunities there are." |
|  |  | Coping with stress | Participants stating that content related to managing stress, such as exercises, tips, and strategies for relaxation, was helpful in reducing stress and promoting well-being, particularly in challenging or uncertain situations. | 12 (29.3) | "When I was stressed about finding a place to live, I kept going back to the app. I looked for advice on dealing with stress, like taking walks or practicing relaxation exercises. These tips helped me a lot in those stressful moments." |
|  |  | Housing | Content related to housing includes information on finding housing, interacting with landlords, the apartment search process, and related rights and duties, especially for newcomers in Switzerland. | 10 (24.4) | "For example, the topic of apartment search, how and where to search for a place. Especially the relationship with the landlord and what to pay attention to during viewings." |
|  |  | Rights, systems, daily life in Switzerland | Participants describe finding content about the Swiss legal system, daily life, and integration particularly helpful in understanding their rights, navigating work and housing, and adjusting to life in Switzerland. | 9 (22.0) | "The app provided many helpful tips, especially about integration in Switzerland, how to interact with locals, and what rules to follow. It helped me understand the Swiss system better." |
|  |  | Social life | Refers to positive feedback on the information and resources provided by the app that help users navigate social interactions, cultural differences, and integration within Swiss society. It includes topics such as how to interact with locals, cultural norms, and building relationships within the community. | 7 (17.1) | "The best part of the app is about how to meet people, your neighbours around you. Primarily, it is the neighbours who are the easiest to get to know in Switzerland." |
|  |  | Health system | Participants giving positive feedback regarding the app's information about the Swiss healthcare system, including access to medical services, insurance, and specific health-related advice. | 6 (14.6) | "The app was particularly helpful with health-related topics, for example, the women's shelter for women's health and household issues. There was so much information I didn’t know before. Today, I have learned more about these topics, like the compensation funds or insurance here, different types of insurance, and debt counselling." |
|  |  | Family reunification | Refers to the positive feedback on information provided by the app regarding the process of bringing family members to Switzerland, particularly for asylum seekers or those with humanitarian visas. | 4 (9.8) | "What I often notice is that people with an F permit often have questions about family reunification. When and how can I bring my family to Switzerland? You only hear that it is impossible or are given false information. The app solves exactly that." |
|  |  | Pain | Participants express that they found content related to pain management, particularly exercises for chronic pain, to be helpful in managing their discomfort and improving their well-being. | 3 (7.3) | "For example, my arm always hurts because I have chronic pain, and the exercises in the app for chronic pain helped me. I tried to implement them, and even though it didn’t help much, it still contributed to me feeling better when I did them." |
|  |  | Sleep | Participants indicate that they found content related to sleep, particularly routines and audio exercises, helpful in improving their sleep quality and health habits. | 3 (7.3) | "For example, I started creating a routine for sleeping and organizing my daily schedule." |
|  | Preferred formats |  |  |  |  |
|  |  | Videos (information and testimonials) | Participants preferred videos, especially testimonials and informational videos, as they provided clear, accessible, and motivational content that was easy to understand and offered real-life experiences. | 16 (39.0) | "What I liked the most were the videos about people sharing their experiences. They provided helpful information and showed how others dealt with their challenges." |
|  |  | Audio-exercises | Participants expressed satisfaction with the audio exercises in the app, finding them helpful for stress reduction, sleep improvement, and general well-being. | 15 (36.6) | "There were many helpful audio exercises that helped with stress, and also information about asylum status, rights in Switzerland, that was good." |
|  |  | Illustrations | Participants express satisfaction with the use of images in the app, appreciating their role in making content easier to understand and more engaging. | 7 (17.1) | "It was easy to use, especially with the pictures. Everything looked so  beautiful." |
|  |  | Written information | Participants preferred written information as it was easier to understand, especially for practical topics like housing, integration, and legal matters. | 5 (12.2) | "For me, reading is better than watching videos. If you have the same content from the videos in separate articles, like reading about someone’s experiences, that would be great." |
|  |  | Written stories from Sui’s neighbourhood | Participants appreciated written stories from the neighbourhood as they provided real-life experiences that helped them relate to others' journeys and challenges. | 4 (9.8) | "I particularly liked the stories about the people, the experiences they had, because it showed what kind of experiences people went through. It felt real." |
|  |  | No preference | Participants indicated no strong preference for a specific format, expressing satisfaction with all available options. | 3 (7.3) | "It was actually all good, everything was fine as it was." |
|  |  | Disliked the illustrations | Participants express dissatisfaction with the images in the app, citing them as superficial, stereotypical, or inappropriate for the app’s context, often preferring more realistic or neutral visuals. | 3 (7.3) | "The images used were not suitable. They seemed stereotypical, like the woman with the headscarf. This creates a political image of Islam,  which I find inappropriate." |
| **Description of app use** |  |  |  |  |  |
|  | Frequency of use |  |  |  |  |
|  |  | Used as needed | Descriptions of the app used on an as-needed basis, with users accessing it when they require specific information or help for particular situations or tasks. | 11 (26.8) | "I used the app about five to six times and each time for about five to ten minutes." |
|  |  | Future use of app | Refers to the participant's intention or plans to continue using the app in the future, often based on specific needs or expectations. | 7 (17.1) | "Yes, for example, if I were to look for a job or learn something new in life, I would definitely use the app. I'm already settled in a place, so I wouldn’t need it for housing, but for other things I would rely on it." |
|  |  | Used rarely | The app is used infrequently (often due to time constraints, technical issues, or a lack of immediate need for the information it provides) | 6 (14.6) | "I have two daughters, many appointments, many doctor visits. I always have to go back and forth, so I only opened the app briefly and then closed it. I have many things to do at home, so I don’t often find time for it." |
|  |  | Used frequently | The app is used frequently, with users engaging with it often (either because they find it essential or because they want to access important information on a regular basis) | 4 (9.8) | "At the beginning, I was very, very active in it. After a while, I started studying, and then I didn’t have as much time, but generally, I used it a  lot." |
| **Content limitations** |  |  |  |  |  |
|  | Unhelpful content |  |  |  |  |
|  |  | Well-being chapters not needed | This subcategory refers to participants who do not find the well-being chapters in the app helpful or relevant to their needs, as they do not experience psychological issues or are already familiar with the information. | 7 (17.1) | "Since I don’t have any psychological issues or stress, I didn’t need to use these parts, but out of curiosity, I checked them out occasionally. Especially the audio exercises, I thought the way they were presented was interesting." |
|  |  | Well-being chapters too superficial | This subcategory refers to participants who find the well-being chapters in the app too superficial, lacking in depth or practical solutions to real-life problems. They feel the content does not address underlying causes or offer actionable steps. | 4 (9.8) | "There are many psychological problems presented in the app, but often no solutions were offered. It would be helpful if solutions were directly linked to the issues." |
|  | Mismatch with needs |  |  |  |  |
|  |  | Missing implementation steps | This subcategory reflects statements where the information provided in the app was considered insufficient for users to take actionable steps in their real-life situations. Users express the need for more practical, step-by-step guidance or assistance in implementing the information they receive, particularly when it comes to housing, legal matters, or integration support. | 12 (29.3) | "In the app, regarding housing, there is a lot of information on what, how, and where you can organize things. These are the initial pieces of information, but what's missing is the next step, how to implement them. I wish I had support with tasks like making appointments, for example, because these things need to be done in German, and someone should help you with that." |
|  |  | Unhelpful for individual cases / too superficial | This subcategory refers to users who find that the app provides generic or superficial information, which doesn't address specific, personal situations. The app lacks detailed guidance or solutions for users' unique cases and often only presents broad, general knowledge that can be easily found elsewhere, without offering deeper insights or practical help. | 8 (19.5) | "For example, there were no concrete details on how I could pursue an apprenticeship. The app only offered very general information, but didn’t provide specific guidance on how to actually start an apprenticeship in my case." |
| **Barriers to use** |  |  |  |  |  |
|  | Subjective reasons |  |  |  |  |
|  |  | Purpose / use unclear | This subcategory includes statements about not having a clear understanding of the app's purpose or did not identify specific goals for using it. | 10 (24.4) | "I didn’t really use the app much because I wasn’t sure what exactly I was supposed to do with it. I just opened it sometimes, but I didn’t have a clear reason for using it." |
|  |  | Not checked everything | This subcategory refers to participants who said that they did not engage with or explore all the features or content available in the app, resulting in limited interaction or incomplete use. | 5 (12.2) | "I haven't looked at everything, but what I did look at was helpful in the app and clear. However, I didn’t explore all of it." |
|  |  | Too little time / too much to do | This subcategory refers to participant's statements about being unable to use the app frequently or fully due to time constraints or a busy schedule with other personal or professional commitments. | 3 (7.3) | "I have two daughters, two small daughters at home, so I don't have much time to use the app. I also filled out the questionnaires, but I didn’t just use the app once, I logged in multiple times and looked at things." |
|  | Technical problems |  |  |  |  |
|  |  | Loading times | Participants reporting having experienced long loading times or slow processing when navigating the app, which affected the overall usability and user experience. | 5 (12.2) | "The only issue was with loading. You always had to wait for it to load." |
|  |  | Login difficulties | Participants expressing experiencing difficulties logging into the app, which caused confusion and frustration regarding its use and purpose. | 4 (9.8) | "I had difficulties logging in due to technical problems. It took a while before I could even get in. Once I was inside, I wasn’t sure how to use it.  I thought I had to wait for instructions before using the app. It was confusing to know what to do once I logged in.'" |
|  | Language difficulties |  |  |  |  |
|  |  | Reading difficulties | Statements of participants that faced challenges understanding the written content in the app, especially due to difficulties with formal Arabic or low literacy levels in their native language. | 5 (12.2) | "I sometimes had trouble understanding certain sentences written in formal Arabic. I mainly learned written language through apps and messages in dialect, so it was hard for me to understand some sentences because I’m not very good at reading." |
| **Suggestions for improvement** |  |  |  |  |  |
|  | Adaptations |  |  |  |  |
|  |  | Collection of words how app would be recommended | This refers to the ways users would recommend the app to others, often highlighting its most useful features or its value for specific situations. | 11 (26.8) | "I would recommend it by saying: It's an Arabic-language app, and it's not in German, but it has many helpful pieces of information. It's very easy to use. As I mentioned before, you don't need a peer to use the app, it's so simple and straightforward that anyone can use it." |
|  |  | First impression: clarity of purpose and more guidance | Participants suggesting that the app could provide a clearer initial overview, explicitly stating its purpose and offering better guidance on navigation and structure to reduce confusion and overwhelm. | 5 (12.2) | "When I opened it for the first time, I didn’t get any tips or information: What is this app for? What can I use in the app? I missed this introductory information. For example, on the homepage, I kept coming back, but I didn’t know where or how to start." |
|  |  | Explanation video at the beginning | This refers to the suggestions that the app should include an introductory video that explains its purpose, features, and how to navigate it effectively. | 5 (12.2) | "Yes, it would definitely help, for example, if there were an introductory video showing how to use the app, what each section is for, and how to navigate through it. That would certainly be very helpful." |
|  |  | Explanation at the beginning | Participants that recommended including an introductory explanation, such as a text or person, at the start of the app to clarify its purpose, features, and navigation options. | 4 (9.8) | "For example, if someone contacted me and explained what’s in the app, what the app is for, and how I could use it, that would definitely be very helpful." |
|  | Additions |  |  |  |  |
|  |  | Links to organisations for counselling | This subcategory includes suggestions for integrating external resources, such as organizations or agencies that provide legal, psychological, or social support, into the app. | 13 (31.7) | "It is important for the app to describe specific places, e.g. existing links, where you can go directly, where you can perhaps also get in touch with people, that would be helpful if that were also available in the app, so not just information, but also the second and third steps." |
|  |  | Information on language acquisition | This subcategory refers to suggestions for providing more specific information and resources related to language acquisition, including practical tips, language courses, and advice on how to improve language skills, particularly in German, for newcomers. | 10 (24.4) | "It would be very helpful if the app offered information about free German courses, where they take place, and how to access them." |
|  |  | Information on leisure activities / social contacts | This subcategory focuses on participants’ suggestions for integrating more detailed, accessible information about leisure and social activities in the app. Suggestions include offering guidance on recreational activities, social events, cultural and sports opportunities, and tips for integrating into the community and engaging with local culture. | 10 (24.4) | "I wish there were more information available on activities and events, especially for people who are new to Switzerland." |
|  |  | Information on financial support / affordable offers | This subcategory includes suggestions for providing information about financial support, affordable offers, or resources for individuals in need, such as assistance for children, healthcare, and general cost-saving opportunities. | 8 (19.5) | "It would be very helpful if I had contacts or addresses for foundations or organizations that could help with the topic of wanting children, as all the tests related to this are very expensive. For example, what was required of me last time cost about 5,000 francs, and it is not covered by health insurance." |
|  |  | More testimonials | This subcategory refers to suggestions for adding more personal experiences and success stories (e.g., videos or written testimonials) to the app, particularly from people who have navigated challenges, such as finding housing or learning the language, to inspire and guide others. | 4 (9.8) | "For example, stories about finding housing, especially personal stories from people, both in video and written formats, with tips on how to find a place to live, would be very helpful." |
|  |  | Information on employment opportunities | This subcategory includes suggestions for improving the app by providing more detailed, practical information about job opportunities, career paths, vocational training, and how to access support and guidance in the Swiss labour market. | 4 (9.8) | "For example, when it comes to work, it would be helpful if the app provided information about professions in Switzerland, including what jobs exist, the pros and cons, and the necessary steps to pursue them. It would be really useful to have a clear overview of what’s available and how to get started" |
|  |  | Regular content updates | This subcategory includes suggestions about the need for regular updates to the app’s content, ensuring the information stays relevant and up-to-date, especially in areas like asylum procedures, peer support, and other crucial topics. | 3 (7.3) | "Well, it depends. If you add new things, sure. Newer people in Switzerland might use it for a longer time, but since I know many things already, it would be more helpful if new topics were added." |
|  |  | Information on culture in Switzerland | This subcategory addresses the suggestions for adding cultural education and advice for newcomers to Switzerland, including guidance on adapting to local norms, values, and practices. | 3 (7.3) | "It would be better if they could give me advice on what makes sense in this context. The situation would be different if I were in Germany or France, but now I’m in Switzerland. I think there are basic requirements for living here." |
|  | Connecting with people |  |  |  |  |
|  |  | Daily support | This subcategory focuses on the expressed need for support in daily life provided through the app, emphasizing the value of connecting with individuals who can provide direct, personalized assistance with practical matters such as navigating the asylum process, housing, and language learning. | 19 (46.3) | "It would be very helpful if the app allowed users to contact someone who could give them specific information or support, especially for practical tasks like setting up appointments or finding a translator for medical visits. Having someone guide them through these steps would make a big difference in their ability to take action." |
|  |  | Contact with Swiss people | This subcategory focuses on the expressed needs for opportunities to connect with Swiss people, including language exchange, social integration, and local community engagement, to help newcomers build relationships and improve their understanding of Swiss culture and daily life. | 13 (31.7) | "For example, it would be helpful if the app provided information about places to go, activities, and how to connect with people in Switzerland. I would like to meet people to practice my German and get to know the country better, not just through messages but in person. I want to build friendships and connect with others who understand the local culture." |
|  |  | Contact with people who speak the same native language | This subcategory includes about wishes of connecting with individuals who speak the same native language, offering a sense of comfort and shared understanding, which can support integration, language learning, and addressing specific needs like legal or cultural questions. | 10 (24.4) | "It would certainly help me if I had someone who has either had exactly the same experience or a specialist who - what's important to me is someone who speaks my language and can support me. Someone who can show me how to beat this path." |
|  |  | Companion within the app | Statements about the importance of having a peer or mentor within the app—someone who has more experience in the country and can offer advice, support, and share useful information to help with integration, legal issues, and general assistance. | 9 (22.0) | "Yes, it would definitely help if there was a way to contact someone in the app who could give me advice, someone who could tell me where to go or direct me to certain organizations. I’ve been looking for a free lawyer or someone who can help me for a long time, and finally, I found someone in a French-speaking canton who tried to help me." |
|  |  | Support from professionals | This subcategory includes wishes for professional support, such as legal, psychological, or other specialized assistance, to help individuals navigate various challenges, ideally within the app. It focuses on having accessible, expert guidance, with the option for personal, one-on-one support. | 7 (17.1) | "It would really help if there were a way to connect with a professional, like a legal advisor or someone who understands these issues. For example, I wanted to bring my sister-in-law from Syria to Switzerland, but the asylum services told me that it's not possible right now. If an organization like the Red Cross or someone else could help me, that would have been very useful." |
|  | Increasing awareness of Sui app |  | This subcategory focuses on reports about the need to increase awareness and visibility of the app, particularly among refugee and migrant communities. Suggestions include making the app available to a wider audience, improving outreach, and using social media to promote it. | 6 (14.6) | This subcategory focuses on the need to increase awareness and visibility of the app, particularly among refugee and migrant communities. Suggestions include making the app available to a wider audience, improving outreach, and using social media to promote it." |
| **Peer support experiences** |  |  |  |  |  |
|  | Concept perceived as helpful |  | Statements that say that peer support could be beneficial. | 6 (14.6) | "Someone sent me an SMS, but I didn’t fully understand it. For example, if someone contacted me and explained to me what the app has, what it’s for, and how I can use it, that would definitely be very helpful." |
|  | Experience and impact |  |  |  |  |
|  |  | Motivated to use the app and stay engaged | This subcategory includes descriptions about how peer support encourages and motivates individuals to engage more actively with the app, often through personalized advice, reminders, and support. | 9 (22.0) | "It was really nice to feel that someone is there to support you, offering help and tips. It made me feel much better and more motivated to continue using the app." |
|  |  | Positive experience | This subcategory describes experiences where individuals had positive interactions with peers, feeling supported, encouraged, or helped through communication, guidance, or motivation. | 8 (19.5) | "It was generally a good experience, especially writing to someone and receiving responses. That was a good experience. The positive part was, there are so many things you don’t want to tell everyone, not even your brother. So, I could interact with him about questions and talk about things I would never discuss with anyone else. That was very helpful." |
|  |  | Unclear use | In this subcategory reflections about situations where participants were unclear about how to use the peer support function are collected. | 6 (14.6) | "Exactly, because I didn't know how to use the app, for example, who I could contact and whether I could contact anyone at all, which confused me a bit." |
|  |  | Peer support not perceived | This subcategory refers to reports where participants did not recognize or were unaware that they had access to peer support, either due to lack of visibility, understanding, or engagement with the service. | 5 (12.2) | "I hadn’t even realized there was a peer." |
|  |  | Positive feelings | This subcategory captures emotional responses of individuals who feel supported, motivated, or cared for during their interactions with peers, leading to positive feelings like gratitude, comfort, or encouragement. | 4 (9.8) | This subcategory captures emotional responses of individuals who feel supported, motivated, or cared for during their interactions with peers, leading to positive feelings like gratitude, comfort, or encouragement. |
|  |  | Motivated to stay in contact | This subcategory refers to reports about the motivation created by the peer's consistent communication, encouraging the individual to maintain regular contact and engage in ongoing dialogue. | 3 (7.3) | "The peer sent messages every week, asked about my health. Then I asked, but how was it exactly for you, and he said: It was really nice, just the fact that there was always someone checking on you or communicating with you, that was always very nice." |
|  | Type of support |  |  |  |  |
|  |  | To ask specific questions and receive recommendations | This subcategory involves users finding it particularly helpful to ask specific questions and receive tailored recommendations or advice from a peer. The interaction focuses on addressing individual needs or challenges, such as navigating a new system, improving well-being, or resolving personal issues, rather than generic or standard responses. | 12 (29.3) | "When I go to a new country, and someone is introduced as a companion, I expect them to be available for my questions, to explain how things work here. I don't need motivational messages once a week but someone who can focus on my questions and my needs, not sending standard messages and then being unavailable." |
|  |  | To receive psychological support | This subcategory includes the comments about the importance of receiving psychological support or tips that help users cope with stress, improve their well-being, or address emotional challenges. Such support often involves empathetic listening, encouragement, or practical advice to enhance mental health. | 4 (9.8) | "At one point, I was under a lot of psychological pressure and couldn’t talk to anyone, not even my family. The peer offered me the opportunity to write to them and share everything I wanted. That helped me the most because, at that time, they were the only person I could talk to about it." |
|  |  | For tips | This subcategory entails comments where peers provided helpful tips, ideas, or practical suggestions to users, which were perceived as valuable in navigating challenges or utilizing the app effectively. | 3 (7.3) | "They always gave me tips and ideas, motivating me to use the app. It was a very good experience." |
|  | Frequency, intensity |  |  |  |  |
|  |  | Every other day | This subcategory describes the mentioned preferences for peer support interactions to occur more frequently than once a week. | 7 (17.1) | "It was disappointing that messages were only sent once a week. That felt too long, and I lost motivation to use the app. But whenever messages came, I felt more motivated. Ideally, someone would message every other day or even daily to show they’re there for me, which would make me feel much more motivated to use the app." |
|  |  | On request | This subcategory includes descriptions of participants' preference for peer interaction to occur only when initiated by the user, ensuring responses are available upon request rather than on a fixed schedule. | 4 (9.8) | "It doesn’t need to be every week that they write on their own, but when I write, I would like them to respond." |
|  | Peer characteristics |  |  |  |  |
|  |  | Experience and expertise in the integration field are important | This subcategory subsumes the wishes of participants about the peer supporters having both professional expertise and experience in the field of integration. | 9 (22.0) | "It is not important that the person has experienced the same thing but that they have enough experience and professional knowledge. They need to analyse and understand my situation and offer concrete solutions. But it doesn't have to be that they went through the same thing as I did. Understanding and expertise are what matter." |
|  |  | Having shared experiences is important | Reports of participants stating an importance of peers having similar lived experiences to the individuals they are supporting. The shared experience allows the peer to empathize with and understand the challenges the individual is facing, providing more effective support. | 4 (9.8) | "It is definitely very helpful if the person has experienced the same thing because they just feel what you’re going through. They can understand when you talk about something because they’ve gone through the same thing." |
| **Daily needs and challenges of participants** |  |  |  |  |  |
|  | Daily life description |  |  |  |  |
|  |  |  |  |  |  |
|  |  | German language course | This subcategory refers to participants' descriptions related to learning German through courses, including their challenges, goals, and suggestions for enhancing the process of language acquisition. | 8 (19.5) | "My everyday life looks like this: I study German, currently at B1 level, and I didn't have much time, but through the SMS reminders, I kept checking and going." |
|  |  | Nothing, staying at home | This subcategory collects reports where participants are limited in their activities and social interactions, often due to legal, health, or personal constraints, leading to periods of inactivity or staying at home. | 6 (14.6) | "My daily life is nothing. I do practically nothing. I don't attend any German courses because I am not allowed to. I am not allowed to work either. Although I have had my two interviews, I still haven't received a decision on my asylum request, and that's why I can't do anything right now." |
|  |  | Childcare | This subcategory collects the statements around the need for support of caring for children. | 5 (12.2) | "I have two small daughters who need intensive care, so I can't, for example, attend courses or do something for myself. I would like to learn something or do something, but right now I am fully occupied with the children." |
|  |  | Housework | This subcategory includes the participants' statements about managing housework, including daily tasks like cooking, cleaning, and organizing home life. | 3 (7.3) | "In the morning, I wake up, send my children to school, and then I go to a course. Around noon, I come back, prepare food for the children, and eat with them." |
|  |  | Job | This subcategory includes participants' experiences, challenges, and needs related to their work life, including employment, job searching, job conditions, or balancing work with other aspects of daily life. | 3 (7.3) | "I have two jobs, I sleep for eight hours, but I sleep long because my work starts at 11 AM with food delivery, and my night job is in logistics, sorting packages. I have a daughter, I’m divorced, I see my daughter twice a week. I try to manage my work and then my daughter visits me." |
|  |  | Life in asylum centre | This subcategory refers to the daily experience of individuals living in asylum centres, which may include dealing with crowded environments, limited activities, and the challenges of maintaining personal and social connections in such settings. | 3 (7.3) | "I wake up every morning, try not to spend too much time in the asylum centre, visit my mother, my brother. I try as much as possible to learn German, to learn the language." |
|  | Practical problems |  |  |  |  |
|  |  | Language | This subcategory captures statements about challenges faced by participants related to language barriers, which hinder their ability to communicate, integrate, and access essential services or opportunities, such as employment and education. | 17 (41.5) | "One of the difficulties I face is that I don’t speak the language. When I try to contact people on the street, I often get shut down. It’s also a form of discrimination. I wish there were opportunities to connect with people, build friendships. This is not available, and that’s very important to me." |
|  |  | Lack of information / advice | This subcategory refers to reports about the lack of necessary or relevant information that individuals need in order to address practical issues they face in daily life. It involves challenges such as missing guidance, unclear instructions, or insufficient access to resources, often leading to confusion or unmet needs. | 17 (41.5) | "I know a lot of people who came here earlier, and they were settled in whatever country, they came there, an apartment was organized for them. Everything was organized and we made sure that they could learn the language as quickly as possible, that they could integrate, that they were supported in their everyday lives. That's what I miss here, because here you're told everywhere: you can go there or go back and forth, but nobody accompanies you. You always have to look after yourself and that's very difficult." |
|  |  | Work / education | This subcategory includes statements about challenges related to accessing employment and education opportunities, including issues such as difficulties in continuing one's profession, barriers to obtaining necessary qualifications, or restrictions on work due to legal or language barriers. | 12 (29.3) | "I was a sports teacher in my home country, and I would have liked information or the chance to continue in that profession. Through my job coaching, I received an offer to teach sports to other refugee women in a restaurant. It helped both me and them a lot, but it couldn't continue due to financial reasons." |
|  |  | Financial situation | This subcategory includes reports on challenges related to financial instability, including issues such as insufficient social assistance, the high cost of services, and difficulties in managing living expenses or supporting family needs. | 11 (26.8) | "There may be certain things, but I can't imagine that an app can change that, for example, that social welfare money, asylum social welfare money, is relatively little, but I can't expect an app to be able to change that." |
|  |  | Housing | This subcategory includes difficulties participants face in securing and managing housing, such as finding available apartments, understanding housing rights and obligations, and lacking support or guidance during the process. | 11 (26.8) | "Especially the topic of finding/looking for an apartment. I'm very preoccupied with that at the moment. We’re in the asylum centre and it's very difficult, especially for the children, when they keep seeing other people finding an apartment and they don't understand why we can't find one. It's quite exhausting and it's also very stressful for me because I have no support at all, no translator, no support with this issue. That's what worries me the most." |
|  |  | Residence status | This subcategory refers to reports about challenges related to an individual's legal status in the country, such as difficulties with obtaining or changing residence permits, work permits, asylum decisions, or citizenship status, as well as the psychological strain caused by uncertainty and lack of clear information. | 8 (19.5) | "Yes, of course. That would be a great thing if you have someone you can ask questions to who is familiar with the Swiss system, with legal issues, with integration issues, for example if I want to change from B to C, then I can ask him, he can tell me what it's like in the canton of Uri, what are the criteria you have to meet." |
|  |  | Unfair system | This subcategory highlights reports of inequality or unfair treatment within the system, particularly related to migration policies, housing, job opportunities, and the lack of individual consideration for refugees. It reflects frustrations about systemic obstacles, discrimination, and the impact of bureaucratic processes. | 8 (19.5) | "I feel a bit like regretting that Switzerland accepted my asylum application at all, because right now, I’m here and I can’t do anything. I see no benefits, it feels like I’m in Africa, but in a clean country. I can't see any advantages." |
|  |  | Denial of skill use | This subcategory refers to the statements about perceived loss of professional or personal skills due to challenges such as migration, lack of recognition of qualifications, and barriers to using previous experience or education in a new environment. | 3 (7.3) | "I was a physical education teacher in my home country, and I would have liked to continue in that field. Through my job coaching, I got the opportunity to teach sports to other refugee women in a restaurant room, and it was beneficial for both me and them. But it couldn’t continue due to financial reasons." |
|  | Socio-cultural problems |  |  |  |  |
|  |  | Integration | This subcategory refers to the challenges participants reported to be facing in integrating into the local community. | 8 (19.5) | "I would have especially liked the opportunity to connect with locals. Despite my health issues, I tried to teach himself the language, but I can't use it because I have no one to talk to. When I try to make contact on the street, he is often shut down. There is also discrimination. The opportunity to meet people and build friendships is simply not available, and that would be very important to him." |
|  |  | Separated family and family reunification | This subcategory collects reports related to family separation and the difficulties of family reunification, including emotional distress due to being apart from family members. | 6 (14.6) | "For example, my family is in the Netherlands, and I can't visit them. At the end of the year, there is a wedding, but I can't attend. I do not receive a residency status or travel documents to go there, and this is psychologically burdensome for me. It makes me very unhappy." |
|  |  | Social isolation | This subcategory refers to the expressed challenges individuals face in forming social connections, leading to feelings of loneliness or isolation. | 7 (17.1) | "I often tried to make connections myself. But so far, I only know one person in Switzerland, which is not normal. Of course, I would be very happy if the app could cover this and provide opportunities or tips on how this should work and if there was a way to contact others through it." |
|  |  | Insufficient support for family here | This subcategory collects reports of participants talking about challenges related to insufficient support for families in Switzerland. | 3 (7.3) | "I have two young daughters who need a lot of intensive care, so I can't, for example, attend courses or do anything for myself. I would like to learn something or do something, but right now, I am fully occupied with the children." |
|  |  | Discrimination | This subcategory addresses reported experiences of unfair treatment based on race, ethnicity, nationality, or personal identity. It includes difficulties in integration, racism, and prejudices faced in daily interactions. | 3 (7.3) | "There is also racism, I have experienced a lot of racism. It happened in the train, on the street, in Zurich city. Yes, with my skin colour, of course, I am Arab and a bit dark, I don't have blue or green eyes. Something strange happened to me: I was on the train and there were two women speaking Swiss German, and they thought I didn't understand German. They said that Arabs, North Africans, Egyptians, and everyone, and everything, that was all. This wasn't the only incident. It also happened in Zurich city, late in the evening." |
|  |  | Taboos around mental health issues | Refers to reports about cultural stigma surrounding mental health issues, especially in certain communities where discussing psychological problems is considered shameful or inappropriate. | 3 (7.3) | "This is especially the case with Arabic-speaking people. If they have psychological problems, they are ashamed of it and would not talk about it. In Switzerland, mental health might not be a problem, and people talk about it, but I also don’t understand: If someone is a little sad and the app shows many diagnoses and hints, 'Oh, you are just depressed.' I think this is not okay because it leads people to believe: 'Oh, I am depressed, oh I have a mental illness.' This can only be said by a psychiatrist or a general practitioner, not an app." |
|  | Psychological problems |  | This subcategory includes reports about the psychological challenges faced by study participants, including stress, depression, anxiety, and other mental health issues affecting their daily lives, emotional well-being, and coping mechanisms. | 10 (24.4) | "For example, my arm always hurts because I have chronic pain there, and in the app, with chronic pain, the exercises helped me." |
| **Quality of life** |  |  | **This category includes only codes from interview guide 1** | *n* = 12 |  |
|  | Subjective assessment |  |  |  |  |
|  |  | Good quality of life | Refers to a positive assessment of one's living conditions, including satisfaction with personal well-being, environment, and social interactions. | 6 (50.0) | "I feel good here, I am working, studying, and I have some friends. It's a normal life, better than the situation I had before." |
|  |  | Rather poor quality of life | Collection of reports about a perception a negative evaluation of one's overall quality of life. | 4 (33.3) | Question: How would you rate your quality of life? Answer: "Zero" |
|  | Meaning of quality of life |  |  |  |  |
|  |  | Switzerland as a beautiful country | This subcategory highlights the reflections about positive aspects of Switzerland, such as its environment, infrastructure, and lifestyle, that contribute to a good quality of life. | 3 (25.0) | "The public transport in Switzerland is good, and the politeness of the people. The country is beautiful." |
|  |  | Stability: financial, work, security, housing, education | This subcategory includes on the key elements mentioned that contribute to a stable and secure life, including financial stability, job security, a safe living environment, and access to education. | 12 (100) | "I do not want to return once the war is over—he wants to build a life here. I have 4 children, with a fifth on the way, and therefore he wants a nice, comfortable life. It is important for me to have a job that he is satisfied with and that is enjoyable. That I don't have psychological or financial pressure when working during the week. I see myself on the path to an ideal life, but as long as I am is still receiving social welfare, it is definitely less comfortable than when I will be independent. The most important thing for me is to earn enough to live well and provide for his family, like paying for housing and health insurance." |
|  |  | Well-being family | This subcategory focuses on reports about the well-being of the family, including the health, happiness, safety, and future prospects of family members. | 11 (91.7) | "Yes, certainly having a regular job, a secure job, having a quiet life, having children who grow up just as I imagine." |
|  |  | Social relationships (partner, children) | This subcategory focuses on the quality of relationships with close family members, particularly partners and children, and how these relationships contribute to overall well-being and life satisfaction. | 11 (91.7) | "The most important thing is that everything is good at home, with my wife, for example, and that I earn enough. Then everything is fine." |
|  |  | Acculturation / language / social contacts | Statements about the process of adapting to a new culture and learning the local language as part of quality of life. | 9 (75.0) | "What's also very important is the language, that's the most important thing because only through language can you integrate into Switzerland." |
|  |  | Psychological health | This subcategory entails reflections of participants' mental and emotional well-being, including stress, emotional pressures, and coping mechanisms as part of a good quality of life. | 7 (58.3) | "Before, the pressure from work often kept me busy. I had taken less care of myself and my health, e.g. eating less. Step by step, I could see an improvement in taking more care of myself. I could better understand what the stress at work is. Before, it was difficult for me to find a balance between work and leisure. By using the app, I was able to learn how to deal with stress. It helped me to find a balance between work and leisure time, e.g. hiking." |
|  |  | Meaningfulness / perspective | This subcategory refers to reports about the sense of purpose, motivation, and future outlook in one's life. It encompasses individuals' aspirations, the importance of contributing to society, and the desire to improve or change their circumstances for a better life. | 5 (41.6) | "I imagine that I don't just want to be a number, but also contribute to society/life. I want to do something meaningful, and specifically, I want to continue studying. That gives me some motivation/hope." |
|  |  | Daily structure | Refers to the statements that the presence of a routine or structured daily life, including regular work, education, or other activities that provide a sense of quality of life | 5 (41.6) | "For me, it is certainly a regulated life. Either you are in training, waking up in the morning, going to training, and coming back, or you work, exactly the same—wake up in the morning and return home in the evening. That is nice, it helps. Unlike me, I currently have nothing to stick to, there is no structured daily life." |
|  |  | Asylum procedure / residence status | Collection of statements that the impact of the asylum process and residence status on an individual’s quality of life is important. | 5 (41.6) | "Most of what hurts and is painful is that I cannot start an education. Because I have been here for a year, and I see how time passes. Everything is lost, I can't use it, but as long as I don't have a residence status, I can't do anything. That is difficult." |
|  |  | Physical health | This subcategory focuses statements on the physical well-being and health of individuals being part of quality of life. | 3 (25.0) | "The most important thing is that one is healthy." |

Notes. n = The number of individuals with at least one coded statement in the respective subcategory. The percentage from the whole sample is shown in brackets. Only subcategories with at least a number of n = 3 were included in this table.

**Appendix D.2-Qualitative Characteristics**

Supplementary Table 5. Demographic data and app usage of participants in the qualitative analysis.

| **Number** | **Age** | **Usage time in minutes** (during 8 weeks after access) | **Gender** | **Study group** (messages from participant to peer) |
| --- | --- | --- | --- | --- |
| 1 | 35 | 2.02 | female | Sui+ (no messages) |
| 2 | 33 | 8.83 | female | WL |
| 3 | 51 | 2.35 | female | Sui |
| 4 | 31 | 30.02 | female | Sui |
| 5 | 36 | 0 | male | Sui+ (no messages) |
| 6 | 19 | 91.73 | female | Sui+ (2 messages) |
| 7 | 28 | 3.35 | male | Sui |
| 8 | 56 | 26.8 | male | Sui |
| 9 | 41 | 22.46 | female | Sui |
| 10 | 44 | 160.07 | male | Sui |
| 11 | 37 | 119.97 | male | Sui+ (5 messages) |
| 12 | 49 | 37.25 | female | Sui+ (no messages) |
| 13 | 57 | 29.42 | female | Sui |
| 14 | 32 | 62.02 | female | Sui+ (2 messages) |
| 15 | 29 | 0 | female | Sui+ (no messages) |
| 16 | 40 | 287.83 | male | Sui |
| 17 | 35 | 13.57 | female | Sui+ (no messages) |
| 18 | 30 | 18.62 | male | Sui |
| 19 | 40 | 235.82 | male | Sui |
| 20 | 40 | 0 | male | WL |
| 21 | 33 | 0 | male | Sui+ (4 messages) |
| 22 | 30 | 0 | male | Sui |
| 23 | 33 | 1.32 | female | Sui |
| 24 | 39 | 34.5 | female | Sui |
| 25 | 37 | 0 | male | WL |
| 26 | 55 | 62.03 | male | Sui |
| 27 | 20 | 0.85 | male | WL |
| 28 | 33 | 17.57 | male | WL |
| 29 | 20 | 34.77 | female | Sui+ (1 message) |
| 30 | 36 | 51.7 | Male | Sui |
| 31 | 40 | 906.0 | female | Sui+ (9 messages) |
| 32 | 37 | 112.6 | male | Sui |
| 33 | 49 | 197.7 | male | Sui+ (4 messages) |
| 34 | 33 | 142.8 | female | Sui+ (11 messages) |
| 35 | 24 | 56.5 | male | Sui |
| 36 | 42 | 70.6 | male | Sui+ (5 messages) |
| 37 | 28 | 12.2 | female | Sui+ (no messages) |
| 38 | 21 | 160.0 | female | Sui+ (14 messages) |
| 39 | 47 | 31.0 | female | Sui+ (no messages) |
| 40 | 28 | 451.8 | male | Sui+ (10 messages) |
| 41 | 38 | 114.0 | male | Sui+ (7 messages) |

Notes. The interviews were conducted more than 8 weeks after participants were given access to the app.
